# Development of Simplified Heterocyclic Acetogenin Analogues as Potent and Selective *Trypanosoma brucei* Inhibitors

**DOI:** 10.1002/cmdc.201600210

**Published:** 2016-06-10

**Authors:** Gordon J. Florence, Andrew L. Fraser, Eoin R. Gould, Elizabeth F. King, Stefanie K. Menzies, Joanne C. Morris, Marie I. Thomson, Lindsay B. Tulloch, Marija K. Zacharova, Terry K. Smith

**Affiliations:** ^1^ EaStCHEM School of Chemistry and School of BiologyBiomedical Sciences Research ComplexUniversity of St. AndrewsNorth HaughSt. AndrewsKY16 9STUK

**Keywords:** [3+2] cycloaddition, drug discovery, natural product analogues, trypanosomatids

## Abstract

Neglected tropical diseases caused by parasitic infections are an ongoing and increasing concern. They are a burden to human and animal health, having the most devastating effect on the world′s poorest countries. Building upon our previously reported triazole analogues, in this study we describe the synthesis and biological testing of other novel heterocyclic acetogenin‐inspired derivatives, namely 3,5‐isoxazoles, furoxans, and furazans. Several of these compounds maintain low‐micromolar levels of inhibition against *Trypanosoma brucei*, whilst having no observable inhibitory effect on mammalian cells, leading to the possibility of novel lead compounds for selective treatment.

Neglected tropical diseases remain one of the largest concerns in the developing countries of Africa and the South Americas, both in terms of healthcare provision and financial impact on the economies of the world′s poorest countries. This ongoing threat has arisen from a lack of effective prevention methods and minimal financial incentive to develop new therapeutic agents.^1^ One of these prevalent neglected tropical diseases which has attracted attention in recent years is African sleeping sickness or human African trypanosomiasis (HAT), caused by the protozoan parasite *Trypanosoma brucei*. HAT is a serious health concern in sub‐Saharan Africa with >65 million people at risk and with an annual mortality rate of approximately 9000 per annum.^2^ The World Health Organization (WHO) estimates 20 000 new cases of HAT per year based on reported cases, and has set an ambitious target to eradicate HAT by 2020.^3^ Current drug treatments depend on the stage of HAT and are difficult to administer to patients, requiring lengthy infusion rates, and have varying degrees of human toxicity, while showing low efficacy toward the parasite.^4^ The combination eflornithine/nifurtimox therapy (NECT) for stage 2 HAT has proven successful, but resistance to this combination therapy is emerging.^5^ The lack of new effective therapeutic agents and the onset of drug resistance highlights the urgent need for the development of novel small molecule inhibitors of *T. brucei* as potential lead compounds in the quest for new and effective treatments for HAT.^6^

Acetogenins are a class of polyketide secondary metabolites isolated from medicinal plants of the *Annonaceae* species, typically found in tropical regions of West Africa and South America.^7^, ^8^ Isolated in 2004 by Laurens et al. from the roots of bush banana plant *Uvaria chamae*, chamuvarinin **1**, displayed high levels of cytotoxicity toward the KB3‐1 cell line (IC_50_=0.8 nm).^9^ In 2011, our research group reported the first total synthesis of this unique tetrahydopyran‐containing acetogenin and found **1** to exhibit unexpected trypanocidal activity in both the bloodstream and procyclic forms of *T. brucei* (Figure 1).^10^, ^11^ Inspired by chamuvarinin, we sought to design a series of simplified acetogenin‐like analogues retaining key structural and stereochemical features of the parent natural product. These simplified analogues were assembled from a pool of readily accessible chiral tetrahydropyran (THP) building blocks via copper‐mediated click chemistry.^12^ These 1,4‐triazole linked analogues, including **2**, maintained high trypanocidal activity with modest selectivity profiles when compared against the human HeLa cell line.

**Figure 1:**
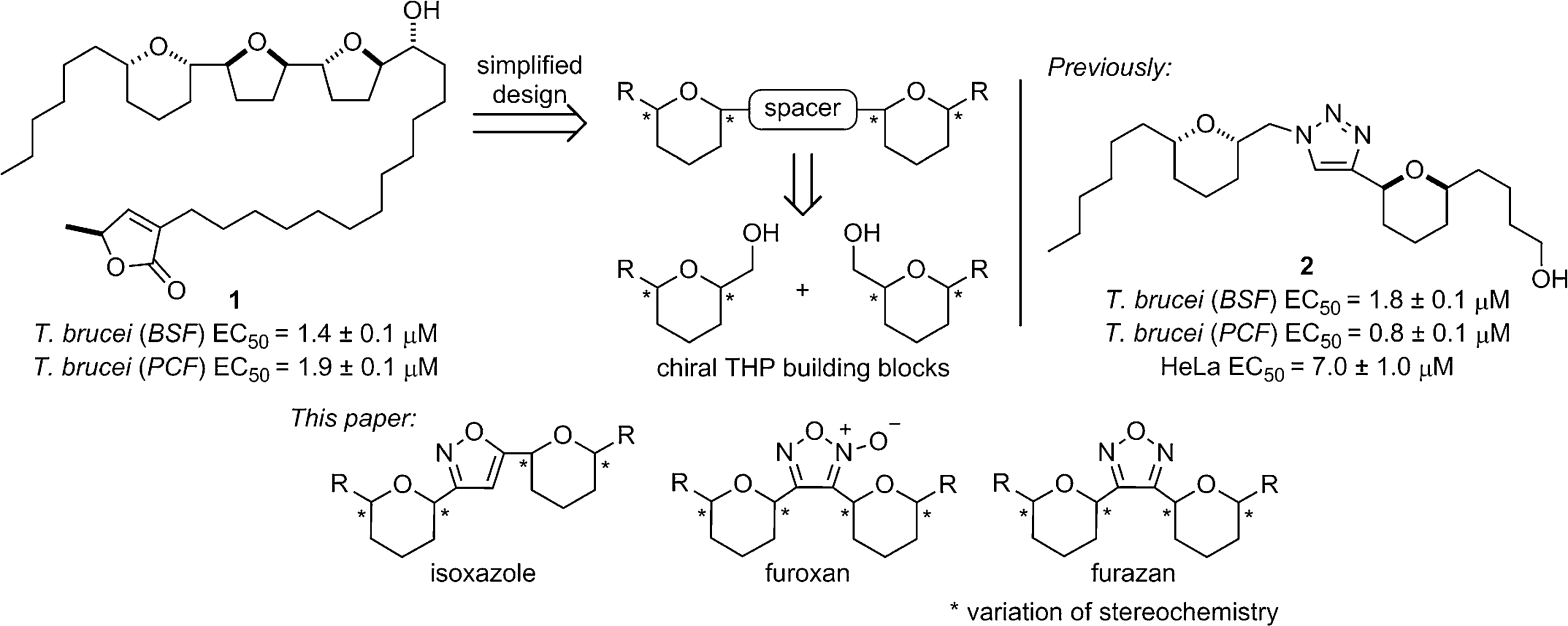
Rationale of new *T. brucei* inhibitors inspired by acetogenins.

Following on from this success we sought to explore alternative heterocyclic linkers, in particular those that would directly attach the heterocycle spacer to the flanking THP rings, in close analogy to the acetogenins. This direct linkage serves to decrease the molecules’ available degrees of freedom and so potentially improve binding efficiency. Moreover, we wished to expand the toolbox of available reactions employing our chiral THP building blocks as a source of molecular diversity. This paper describes the expansion of our methodology to new heterocycles: 3,5‐isoxazoles, furoxans and furazans, as well as their assessment as potential trypanocidal agents.

Despite their prevalence in natural products and their presence in several important drug compounds (e.g., valdecoxib, leflunomide, cloxacillin),^13^ synthetic routes to aliphatic isoxazoles remain extremely limited.^14^ In particular, there are only limited examples of α‐oxygenated 3,5‐isoxazoles and none of these, to our knowledge, have been prepared in enantio‐enriched form. Our approach employs the coupling of chiral α‐oxygenated alkynes with in situ prepared nitrile oxides, derived from the corresponding oximes, in a [3+2] cycloaddition. Oximes **3**–**5** were rapidly accessed from the corresponding THP alcohols by Swern oxidation and condensation with hydroxylamine (Scheme 1). Synthesis of the required alkyne‐substituted THP precursors **6**–**8** have been previously described.^10^, ^12^

**Scheme 1:**
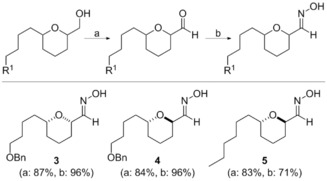
Synthesis of oximes **3**–**5**: a) (COCl)_2_, DMSO, CH_2_Cl_2_, Et_3_N, −78 °C→RT; b) NH_2_OH⋅HCl, EtOH, 0 °C→RT.

A screen of isoxazole‐forming reaction conditions, focused on the choice of oxidising agent (required to generate the nitrile oxide) found that *tert*‐butylhypochlorite was essential as the oxidising agent.^15^, ^16^ These conditions were successfully applied across a range of substrates with varying side‐chain substitution and THP stereochemistry giving isoxazoles **9**–**13** (Scheme 2). Yields were generally modest, but nonetheless rapidly generated sufficient quantities of these synthetically demanding substrates for biological evaluation.

**Scheme 2:**
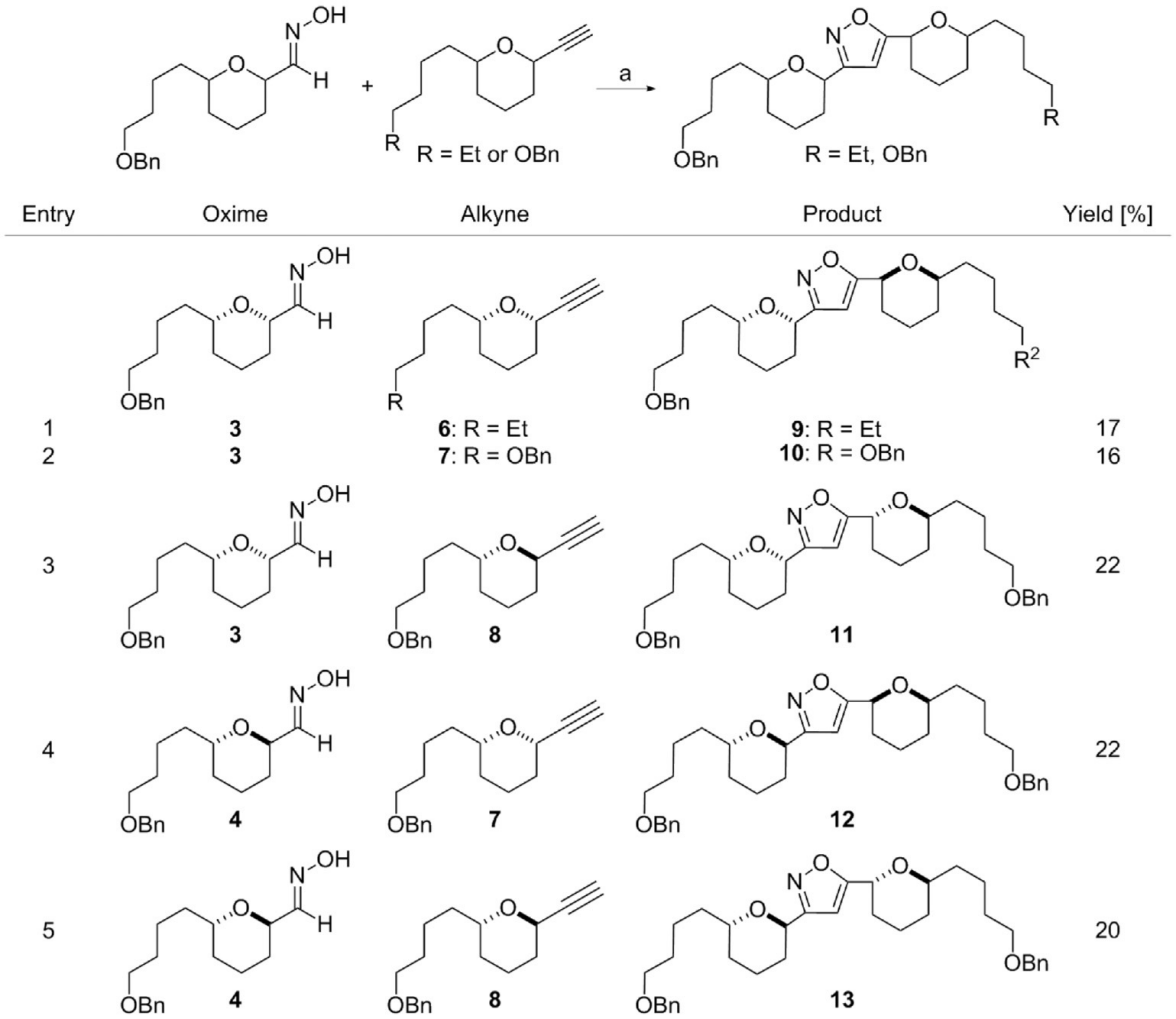
Synthesis of isoxazoles **9**–**13**: a) *t*BuOCl then Et_3_N, −78 °C→RT.

Screening results for our compounds against procyclic and bloodstream forms of *T. brucei* are outlined in Table 1. Also included are the results of HeLa cell line screening which we have employed as a representative human cell line to assess our analogues’ selectivity. In general, the isoxazole motif was well tolerated with comparable activity toward *T. brucei* being maintained to the original triazole analogues. The THP stereochemistry had a significant influence on potency with the *syn–syn* compounds **9** and **10** and *anti–anti* **13** most potent. This is in contrast to our original triazole series where *anti–anti* analogues were generally inactive.^12^ Of particular interest were the good levels of selectivity displayed across the series against *T. brucei* over mammalian HeLa cells, with all but one of the analogues greater than 100 μm against HeLa cells.

**Table 1:** Biological data for isoxazoles **9**–**13**.

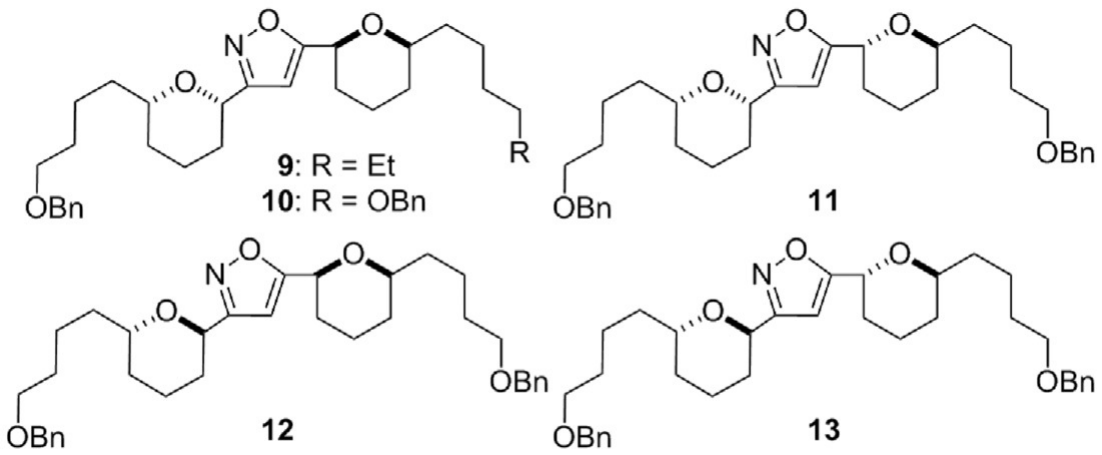					
Entry	Analogue	EC_50_ [μm]			SI^[a]^
		*T. brucei* (BSF)	*T. brucei* (insect)	HeLa	
1	**9**	4.5±0.2	19.6±3.1	107.8±4.9	24
2	**10**	9.0±0.6	43.8±4.0	>200	>22
3	**11**	30.9±1.4	69.3±5.1	>200	>6.5
4	**12**	17.4±0.5	>60	68.8±6.8	4.0
5	**13**	5.6±0.2	16.6±2.2	>100	>18

[a] Selectivity index: (EC_50_ HeLa)/(EC_50_ *T. brucei*).

In the preparation of the isoxazole analogues, we identified significant by‐product formation‐ namely the furoxan arising from nitrile oxide dimerisation.^17^ These could be isolated in trace amounts but were more productively generated by simply warming the reaction after nitrile oxide formation in order to induce dimerisation (in the absence of alkyne). This generated furoxans **14**–**16** in useful quantities (Scheme 3). While interesting in their own right, these compounds also allowed rapid access to furazans **17**–**19** by simple reduction with triethyl phosphite.

**Scheme 3:**
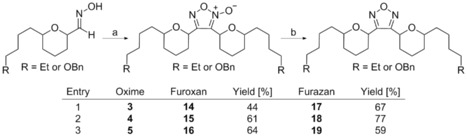
Synthesis of furoxans **14**–**16** and furazans **17**–**19**: a) *t*BuOCl then Et_3_N, −78 °C→RT; b) P(OEt)_3_, reflux.

Screening of these compounds revealed that furoxans also displayed encouraging *T. brucei* activity with *anti–anti* analogue **15** most potent and 5.6 times more selective over HeLa cells (Table 2, entry 2).^18^ Pleasingly, select furazan compounds maintain good *T. brucei* inhibition, while being essentially inactive toward HeLa (**18**, >44‐fold selectivity, entry 5). The selectivity observed in this instance merits further detailed study of this unusual heterocyclic framework.

**Table 2:** Biological data for furoxans **14**–**16** and furazans **17**–**19**.

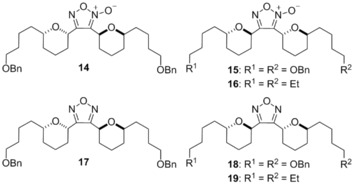					
Entry	Analogue	EC_50_ [μm]			SI^[a]^
		*T. brucei* (BSF)	*T. brucei* (insect)	HeLa	
1	**14**	8.7±0.4	>50	66.6±6.8	7.7
2	**15**	4.5±0.3	5.5±0.2	25.4±12.6	5.6
3	**16**	63.8±5.3	>500	>500	>7.8
4	**17**	16.3±0.5	26.6±1.9	142.9±17.3	8.8
5	**18**	11.3±0.6	>100	>500	>44
6	**19**	146±7.7	>500	>500	>3.4

[a] Selectivity index: (EC_50_ HeLa)/(EC_50_ *T. brucei*).

We have demonstrated the utility of our chiral THP building blocks by their elaboration to oximes and their use in ‘Click’ reactions with alkynes to generate complex isoxazole products. Moreover, dimerisation of these oximes allows access to furoxan and furazan heterocycles. All series tested show encouraging low micromolar activity in *T. brucei* and excellent selectivity over mammalian cells in certain cases. These selectivities are a significant improvement over our previously described triazole compounds and can serve as a basis for further optimisation. Current efforts are focused on other heterocyclic spacers as well as the synthesis of fluorescent and affinity tagged versions in order to isolate target protein(s) allowing us to establish the trypanocidal mode of action.

## Supporting information

As a service to our authors and readers, this journal provides supporting information supplied by the authors. Such materials are peer reviewed and may be re‐organized for online delivery, but are not copy‐edited or typeset. Technical support issues arising from supporting information (other than missing files) should be addressed to the authors.

Supplementary

## References

[1] L. Zhou , G. Stewart , E. Rideau , N. J. Westwood , T. K. Smith , J. Med. Chem. 2013, 56, 796–806. 23281892 10.1021/jm301215e PMC3579312

[2] R. Lozano , M. Naghavi , K. Foreman , S. Lim , K. Shibuya , V. Aboyans et al., Lancet 2012, 380, 2095–2128. 23245604 10.1016/S0140-6736(12)61728-0 PMC10790329

[3] *Trypanosomiasis*, *African*, World Health Organization, Geneva, **2016**, www.who.int/topics/trypanosomiasis_african/en/ (accessed April 7, 2016).

[4a] D. Steverding , Parasites Vectors 2010, 3, 15; 20219092 10.1186/1756-3305-3-15 PMC2848007

[4b] V. Delespaux , H. P. de Koning , Drug Resist. Updates 2007, 10, 30–50. 10.1016/j.drup.2007.02.004 17409013

[5] G. Priotto , S. Kasparian , W. Mutombo , D. Ngouama , S. Ghorashian , U. Arnold , S. Ghabri , E. Baudin , V. Buard , S. Kazadi-Kyanza , M. Ilunga , W. Mutangala , G. Pohlig , C. Schmid , U. Karunakara , E. Torreele , V. Kande , Lancet 2009, 374, 56–64. 19559476 10.1016/S0140-6736(09)61117-X

[6a] P. M. Njogu , E. M. Guantai , E. Pavadai , K. Chibale , ACS Infect. Dis. 2016, 2, 8–31; 27622945 10.1021/acsinfecdis.5b00093

[6b] A. S. Nagle , S. Khare , A. B. Kumar , F. Supek , A. Buchynskyy , C. J. N. Mathison , N. K. Chennamaneni , N. Pendem , F. S. Buckner , M. H. Gelb , V. Molteni , Chem. Rev. 2014, 114, 11305–11347. 25365529 10.1021/cr500365f PMC4633805

[7a] F. Q. Alali , X. X. Liu , J. L. McLaughlin , J. Nat. Prod. 1999, 62, 504–540; 10096871 10.1021/np980406d

[7b] M. C. Zafra-Polo , B. Figadère , T. Gallardo , J. R. Tormo , D. Cortes , Phytochemistry 1998, 48, 1087–1117;

[7c] M. C. Zafra-Polo , M. C. González , E. Estornell , S. Sahpaz , D. Cortes , Phytochemistry 1996, 42, 253–271; 8688168 10.1016/0031-9422(95)00836-5

[7d] L. Zeng , Q. Ye , N. H. Oberlies , G. Shi , Z. M. Gu , K. He , J. L. McLaughlin , Nat. Prod. Rep. 1996, 13, 275–306; 8760865 10.1039/np9961300275

[7e] J. K. Rupprecht , Y. H. Hui , J. L. McLaughlin , J. Nat. Prod. 1990, 53, 237–278. 2199608 10.1021/np50068a001

[8] A. Bermejo , B. Figadère , M. C. Zafra-Polo , I. Barrachina , E. Estornell , D. Cortes , Nat. Prod. Rep. 2005, 22, 269–303. 15806200 10.1039/b500186m

[9a] D. Fall , R. A. Duval , C. Gleye , A. Laurens , R. Hocquemiller , J. Nat. Prod. 2004, 67, 1041–1043; 15217292 10.1021/np030521a

[9b] S. Derbré , E. Poupon , C. Gleye , R. Hocquemiller , J. Nat. Prod. 2007, 70, 300–303. 17279796 10.1021/np060376b

[10a] G. J. Florence , J. C. Morris , R. G. Murray , J. D. Osler , R. R. Vanga , T. K. Smith , Org. Lett. 2011, 13, 514–517; 21174397 10.1021/ol1028699 PMC3031177

[10b] G. J. Florence , J. C. Morris , R. G. Murray , R. R. Vanga , J. D. Osler , T. K. Smith , Chem. Eur. J. 2013, 19, 8309–8320. 23630031 10.1002/chem.201204527

[11] For investigations into the trypanocidal activity of the acetogenins, see:

[11a] S. Sahpaz , C. Bories , P. M. Loiseau , D. Cortès , R. Hocquemiller , A. Laurens , A. Cavé , Planta Med. 1994, 60, 538–540; 7809207 10.1055/s-2006-959566

[11b] A. I. Waechter , G. Yaluff , A. Inchausti , A. Rojas de Arias , R. Hocquemiller , A. Cavé , A. Fournet , Phytother. Res. 1998, 12, 541;

[11c] S. Hoet , F. Opperdoes , R. Brun , J. Quetin-Leclercq , Nat. Prod. Rep. 2004, 21, 353–364. 15162223 10.1039/b311021b

[12] G. J. Florence , A. L. Fraser , E. R. Gould , E. F. King , S. K. Menzies , J. C. Morris , L. B. Tulloch , T. K. Smith , ChemMedChem 2014, 9, 2548–2556. 25145275 10.1002/cmdc.201402272 PMC4298241

[13] A. I. Kotyatkina , V. N. Zhabinsky , V. A. Khripach , Russ. Chem. Rev. 2001, 70, 641–653.

[14a] F. Hu , M. Szostak , Adv. Synth. Catal. 2015, 357, 2583–2614;

[14b] M. Benltifa , S. Vidal , B. Fenet , M. Msaddek , P. G. Goekjian , J. P. Praly , A. Brunyánszki , T. Docsa , P. Gergely , Eur. J. Org. Chem. 2006, 4242–4256;

[14c] R. O. Gould , K. E. McGhie , R. M. Paton , Carbohydr. Res. 1999, 322, 1–13;

[14d] K. V. Gothelf , K. A. Jørgensen , Chem. Rev. 1998, 98, 863–909.11848917

[15] A variety of oxidising agents were screened without success, namely NaOCl, NCS, PIFA, Chloramine-T.

[16] J. W. Bode , E. M. Carreira , J. Am. Chem. Soc. 2001, 123, 3611–3612. 11472140 10.1021/ja0155635

[17] J. Boström , A. Hogner , A. Llinàs , E. Wellner , A. T. Plowright , J. Med. Chem. 2012, 55, 1817–1830. 22185670 10.1021/jm2013248

[18] S. D. Jorge , M. Ishii , F. Palace-Berl , A. K. Ferreira , P. L. de Sá Júnior , A. A. de Oliveira , I. Y. Sonehara , K. F. M. Pasqualoto , L. C. Tavares , MedChemComm 2012, 3, 824–828.

