# Supplementary material for: Development of Simplified Heterocyclic Acetogenin Analogues as Potent and Selective Trypanosoma brucei Inhibitors
Source: ChemMedChem. 2016 Jun 10;11(14):1503–6. doi: 10.1002/cmdc.201600210 (PMC5111590; doi:10.1002/cmdc.201600210)
Supplement: Supplementary file 1 — Supplementary [file CMDC-11-1503-s001.pdf]

CHEMMEDCHEM
Chemistry & Drug Discovery

Supporting Information

Development of Simplified Heterocyclic Acetogenin Analogues as Potent and Selective *Trypanosoma brucei* Inhibitors

Gordon J. Florence,* Andrew L. Fraser, Eoin R. Gould, Elizabeth F. King, Stefanie K. Menzies, Joanne C. Morris, Marie I. Thomson, Lindsay B. Tulloch, Marija K. Zacharova, and Terry K. Smith[a]

cmdc_201600210_sm_miscellaneous_information.pdf

General experimental

Analytical thin layer chromatography was carried out on Merck Kieselgel 60 F254 plates with visualization by ultraviolet light (254 nm) and/or heating the plate after staining with either a solution of 20% ceric ammonium molybdate w/v in H_2O or 20% potassium permanganate w/v in H_2O.

Optical rotations were measured with a Perkin-Elmer 341 polarimeter at 589 nm (sodium D line) and concentrations (c) are reported in g/100 mL.

Infrared (IR) spectroscopy were recorded on a Shimadzu IRAffinity-1 fourier transform IR spectrophotometer using Pike MIRacle ATR accessory. Analysis was carried out using Shimadzu IRsolution v1.50. Wavelengths of maximum absorbance (vmax) are quoted in cm^{-1}. Only selected, characteristic IR absorption data are provided for each compound.

NMR Spectroscopy were recorded using deuterochloroform ($CDCl_3$) as the solvent. Chemical shifts (δ) are given in parts per million (ppm) from tetramethylsilane (δ = 0) and were measured relative to the signal of the solvent in which the sample was analyzed ($CDCl_3$: δ 7.26, ^1H NMR; δ 77.0, ^{13}C NMR). Coupling constants (*J* values) are given in Hertz (Hz) and are reported to the nearest 0.1 Hz. ^1H NMR spectral data are tabulated in the order: number of protons, multiplicity (br, broad; s, singlet; d, doublet; dd, doublet of doublets; t, triplet; q, quartet; m, multiplet), coupling constant and proton assignment using the numbering shown.

Mass Spectroscopy were recorded using a Thermo Fisher LTQ Orbitrap XL mass spectrometer or a Waters Xevo G2-S mass spectrometer by the EPSRC National Mass Spectrometry service (Swansea) using Electrospray Ionization (ES) or Atmospheric Solids Analysis Probe (ASAP) techniques. Other spectra were recorded on a Thermo Exactive Orbitap mass spectrometer using ES techniques at the University of St Andrews Mass Spectrometry service (School of Chemistry and Biomolecular Sciences).

General procedure A: Swern oxidation

To a solution of dimethyl sulfoxide (2.4 eq) in CH_2Cl_2 at -78 °C was added oxalyl chloride (2.0 eq). The reaction mixture was stirred at this temperature for 30 min before the addition of alcohol (1 eq) as a solution in CH_2Cl_2 *via* cannula. The reaction was stirred for a further 1 h, before the dropwise addition of Et_3N (7 eq) and the reaction mixture was warmed to RT over 30 min. After the addition of saturated aqueous NH_4Cl, the organics were

extracted with CH_2Cl_2 (x 3) and the combined organic extracts were dried (Na_2SO_4), filtered and concentrated *in vacuo*.

General procedure B: Formation of oxime

To a solution of aldehyde (1 eq) in EtOH at 0 °C was added a solution of hydroxylamine.HCl (2 eq) in water. After completion of the reaction, the mixture was quenched by the addition of H_2O. The organics were extracted with CH_2Cl_2 (x 3), washed with brine, dried ($NaSO_4$) and concentrated *in vacuo* to afford the desired oxime.

General procedure C: *in situ* formation of nitrile oxide and subsequent cycloaddition (with alkyne partner or dimerisation)

To a solution of oxime (1 eq) in CH_2Cl_2 at -78 °C was slowly added *t*-BuOCl (1.05 eq) and the reaction monitored for oxime consumption by TLC (approximately 5 min). For isoxazole formation, a solution of Et_3N (1.5 eq) and alkyne (2eq) in CH_2Cl_2 (1 mL) was then added. For furoxan formation, only Et_3N (1.5 eq) was added. The mixture was then allowed warm to RT. After 16 h, the reaction mixture was quenched by addition of saturated aqueous NH_4Cl and extracted with CH_2Cl_2 (x 3). The organics were washed with brine, dried (Na_2SO_4) and concentrated *in vacuo*.

Experimental Details

The synthesis of **6**-**8** have been described previously.[1]

***t*-BuOCl**

A mixture of *t*-butanol (*t*-BuOH) (8.00 ml, 83.6 mmol) and glacial acetic acid (5.20 ml, 92.8 mmol) was added to a vigorously stirred solution of sodium hypochlorite (NaOCl) (112 ml, ~1.08 M solution, 120 mmol) at 0 °C. The reaction mixture was left to stir for 14 min in the dark, before separating the organic layer, washing with brine (20 ml) and drying over oven-dried calcium chloride ($CaCl_2$). After filtering over glass wool (cloudy yellow solution), the product was stored in the fridge in an amber bottle over oven-dried $CaCl_2$. No further purification and no characterisation were necessary.

(2*S*,6*S*)-6-[(4-benzyloxy)butyl]-2-[hydroxyimino]tetrahydropyran 3

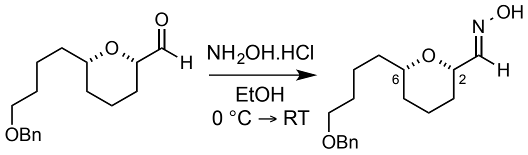

General procedure B was followed with hydroxylamine.HCl (14.7 mg, 0.21 mmol) in water (1 mL) added to the *syn*-THP aldehyde[1] (29.3 mg, 0.11 mmol) in EtOH (2 mL) to afford oxime **3** (28.9 mg, 94%) without the need for further purification.

R$_f$ 0.32 (20% EtOAc/hexane); $[\alpha]_D^{20}$ –22.1 (*c* 1.82, $CHCl_3$); **IR** (thin film) 3335, 2936, 2860, 1454 cm^{-1}; **^{1}H NMR** (500 MHz, $CDCl_3$) δ 7.40 (1H, br s, $\underline{H}_1$), 7.36-7.28 (5H, m, Ar$\underline{H}$), 4.50 (2H, s, OC$\underline{H}_2$Ar), 3.99 (1H, ddd, *J* = 11.3, 5.8, 2.1 Hz, $\underline{H}_2$), 3.45 (2H, t, *J* = 6.6 Hz, $\underline{H}_{10}$), 3.37-3.32 (1H, m, $\underline{H}_6$), 1.90-1.85 (1H, m, $\underline{H}_{4a}$), 1.69-1.39 (10H, m, $\underline{H}_3$ + $\underline{H}_{4b}$ + $\underline{H}_{5a}$ + $\underline{H}_{7\text{-}9}$), 1.28-1.18 (1H, m, $\underline{H}_{5b}$); **^{13}C NMR** (125 MHz, $CDCl_3$) δ 152.1, 138.6, 128.3, 127.6, 127.5, 78.0, 75.1, 72.9, 70.3, 36.1, 30.9, 29.7, 29.4, 23.0, 22.1; **HRMS** (ES^+) Calc. for $C_{17}H_{26}O_3N$ $[M+H]^+$ 292.1907, found 292.1905.

(2*R*,6*S*)-6-[(4-benzyloxy)butyl]-2-(hydroxyimino)tetrahydropyran 4

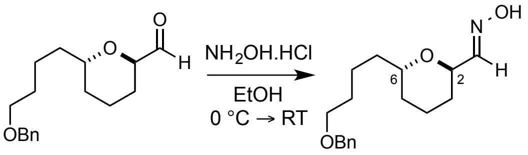

General procedure B was followed with hydroxylamine.HCl (155 mg, 2.23 mmol) in water (11 mL) added to *anti*-THP aldehyde[1] (295 mg, 1.07 mmol) in EtOH (20 mL) to afford oxime **4** (298 mg, 96%) as a colourless oil which was used without further purification. (A sample was purified by flash chromatography (5% EtOAc/hexane) to provide material for characterisation.)

R$_f$ 0.32 (20% EtOAc/hexane); $[\alpha]_D^{20}$ +5.0 (*c* 1.2, $CHCl_3$); **IR** (thin film) 3325, 2932, 2862, 1450 cm^{-1}; **^{1}H NMR** (300 MHz, $CDCl_3$) δ 7.40-7.16 (5H, m, Ar$\underline{H}$), 7.34 (1H, d, *J* = 1.6 Hz, $\underline{H}_1$), 4.54-4.45 (1H, m, $\underline{H}_2$), 4.50 (2H, s, OC$\underline{H}_2$Ar), 3.64-3.51 (1H, m, $\underline{H}_6$), 3.47 (2H, t, *J* = 6.6 Hz, $\underline{H}_{10}$), 1.95-0.86 (12H, m, $\underline{H}_3$ + $\underline{H}_4$ + $\underline{H}_5$ + $\underline{H}_{7\text{-}9}$); **^{13}C NMR** (75 MHz, $CDCl_3$) δ 152.9, 139.0, 128.7, 128.1, 127.9, 73.3, 72.9, 70.7, 70.5, 35.3, 31.0, 30.1, 27.3, 22.6, 19.3; *m/z* **HRMS** (ES^+) Calc. for $C_{17}H_{26}O_3N$ $[M+H]^+$ 292.1907, found 292.1909.

(2*R*,6*S*)-6-hexyl-2-(hydroxyimino)tetrahydropyran 5

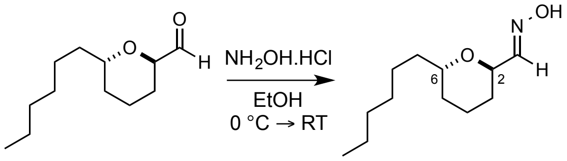

General procedure B was followed with hydroxylamine.HCl (84.0 mg, 1.21 mmol) in water (2 mL) added to *anti*-THP aldehyde[1] (120 mg, 0.605 mmol) in EtOH (3 mL). Purification by flash chromatography (15% Et_2O/hexane) provided oxime **5** (91.0 mg, 71%, 77:23 *E*:*Z*) as a colourless oil.

R$_f$ 0.19 & 0.28 (20% Et_2O/hexane); $[\alpha]_D^{20}$ –10.0 (*c* 0.7, $CHCl_3$); **IR** (thin film) 3327, 2928, 2857, 1458, 1439 cm^{-1}; **^{1}H NMR** (300 MHz, $CDCl_3$), 77:23 *E*:*Z*) δ 8.07 (1H, br s, O$\underline{H}$, *Z*), 7.56 (1H, d, *J* = 3.9 Hz, $\underline{H_1}$, *E*), 7.49 (1H, br s, O$\underline{H}$, *E*), 7.01 (1H, d, *J* = 4.0 Hz, $\underline{H_1}$, *Z*), 4.97 (1H, q, *J* = 4.7 Hz, $\underline{H_2}$, *Z*), 4.52 (1H, q, *J* = 4.0 Hz, $\underline{H_2}$, *E*), 3.76-3.70 (1H, m, $\underline{H_6}$, *Z*), 3.63-3.57 (1H, m, $\underline{H_6}$, *E*), 3.47 (2H, t, *J* = 6.6 Hz, $\underline{H_{10}}$), 1.86-1.53 (6H, m, $\underline{H_{3\text{-}5}}$, *E* & 6H, m, $\underline{H_{3\text{-}5}}$, *Z*), 1.45-1.30 (10H, m, $\underline{H_{7\text{-}11}}$, *E* & 10H, m, $\underline{H_{7\text{-}11}}$, *Z*), 0.90 (3H, t, *J* = 6.6 Hz, $\underline{H_{12}}$, *E* & 3H, t, *J* = 6.6 Hz, $\underline{H_{12}}$, *Z*); **^{13}C NMR** (75 MHz, $CDCl_3$) δ 152.7 (*E* & *Z*), 72.9 (*E*), 72.8 (*Z*), 70.3 (*E* & *Z*), 35.2 (*E*), 34.5 (*Z*), 32.0 (*E* & *Z*), 30.8 (*E*), 30.5 (*Z*), 29.5 (*E* & *Z*), 27.6 (*Z*), 27.1 (*E*), 25.7 (*Z*), 25.6 (*E*), 22.8 (*E* & *Z*), 19.6 (*Z*), 19.0 (*E*), 14.2 (*E* & *Z*); *m/z* **HRMS** (ES$^+$) Calc. for $C_{12}H_{24}O_2N$ $[M+H]^+$ 214.1802, found 214.1801.

3-[(2*S*',6*S*')-6'-(4-(benzyloxy)butyl)tetrahydropyran-2-yl]-5-[(2*S*'',6*S*'')-6''-hexyltetrahydropyran-2-yl)]-1,2-oxazole 9

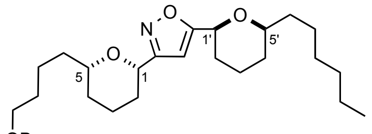

General procedure C was followed with *t*-BuOCl (7.30 µL, 67.0 µmol) added to a solution of oxime **3** (18.5 mg, 63.0 µmol) in CH_2Cl_2 (1 mL) followed by alkyne **6** (25.0 mg, 130 µmol) and Et_3N (13.0 µL, 95.0 µmol) in CH_2Cl_2 (1 mL). Purification by flash column chromatography (5% EtOAc/hexane) provided isoxazole **9** (5.30 mg, 17%) as a colourless oil.

R$_f$ 0.67 (20% EtOAc/hexane); $[\alpha]_D^{20}$ –44.2 (*c* 0.53, $CHCl_3$); **IR** (thin film) 2938, 2860, 1734, 1605, 1454 cm^{-1}; **^{1}H NMR** (300 MHz, $CDCl_3$) δ 7.37-7.28 (5H, m, Ar$\underline{H}$), 6.24 (1H, s, Ar$\underline{H}$), 4.55-4.47 (2H, m, $\underline{H_1}$ + $\underline{H_{1'}}$), 4.49 (2H, s, OC$\underline{H_2}$Ar), 3.51-3.39 (2H, m, $\underline{H_5}$ + $\underline{H_{5'}}$), 3.47 (2H, t, *J* = 6.5 Hz, $\underline{H_9}$), 1.96-1.86 (4H, m, $\underline{H_{2a}}$ + $\underline{H_{2a'}}$ + $\underline{H_{3a}}$ + $\underline{H_{3a'}}$), 1.68-1.53 (8H, m,

$\underline{H_{2b}}$ + $\underline{H_{2b'}}$ + $\underline{H_{3b}}$ + $\underline{H_{3b'}}$ + $\underline{H_{4a}}$ + $\underline{H_{4a'}}$ + $\underline{H_8}$), 1.52-1.38 (4H, m, $\underline{H_6}$ + $\underline{H_{6'}}$), 1.36-1.21 (12H, m, $\underline{H_{4b}}$ + $\underline{H_{4b'}}$ + $\underline{H_7}$ + $\underline{H_{7'-10'}}$), 0.88 (3H, t, J = 6.9 Hz, $\underline{H_{11'}}$); **^{13}C NMR** (125 MHz, $CDCl_3$) δ 172.8, 165.3, 138.6, 128.4, 127.7, 127.5, 98.9, 78.7, 78.4, 72.9, 72.8, 72.7, 70.4, 36.4, 36.2, 31.8, 31.2, 31.0, 30.2, 29.8, 29.4, 25.4, 23.4, 23.3, 22.7, 22.1, 14.1; **HRMS** (ES^+) Calc. for $C_{30}H_{46}O_4N$ $[M+H]^+$ 484.3421, found 484.3414.

3,5-bis(2S,6S)-6-[4-(benzyloxy)butyl]tetrahydropyranyl)isoxazole 10

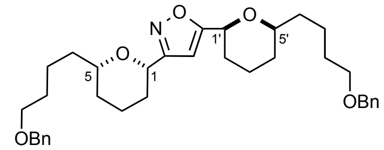

General procedure C was followed with *t*-BuOCl (14.6 µl, 134 µmol) added to a solution of oxime **3** (37.0 mg, 130 µmol) in CH_2Cl_2 (1 mL) followed by alkyne **7** (50.0 mg, 180 µmol) and Et_3N (26.0 µl, 190 µmol) in CH_2Cl_2 (1 mL). Purification by flash column chromatography (5% EtOAc/hexane) provided isoxazole **10** (11.5 mg, 16%) as a colourless oil.

R_f 0.41 (20% EtOAc/hexane); $[\alpha]_D^{20}$ –34.3 (*c* 1.2, $CHCl_3$); **IR** (thin film) 2941, 2857, 2801 cm^{-1}; **^{1}H NMR** (300 MHz, $CDCl_3$) δ 7.35-7.25 (10H, m, Ar$\underline{H}$), 6.24 (1H, s, Ar$\underline{H}$), 4.54-4.49 (2H, m, $\underline{H_1}$ + $\underline{H_{1'}}$), 4.50 (2H, s, OC$\underline{H_2}$Ar), 4.49 (2H, s, OC$\underline{H_2}$Ar), 3.48-3.42 (2H, m, $\underline{H_5}$ + $\underline{H_{5'}}$), 3.47 (2H, t, J = 6.5 Hz, $\underline{H_{9'}}$), 3.46 (2H, t, J = 6.6 Hz, $\underline{H_9}$), 1.95-1.87 (4H, m, $\underline{H_{2A}}$ + $\underline{H_{2A'}}$ + $\underline{H_{3A}}$ + $\underline{H_{3A'}}$), 1.67-1.26 (20H, m, $\underline{H_{2B}}$ + $\underline{H2_{B'}}$ + $\underline{H3_B}$ + $\underline{H3_{B'}}$ + $\underline{H_4}$ + $\underline{H_{4'}}$ + $\underline{H_6}$ + $\underline{H_{6'}}$ + $\underline{H_7}$ + $\underline{H_{7'}}$ + $\underline{H_8}$ + $\underline{H_{8'}}$); **^{13}C NMR** (75 MHz, $CDCl_3$) δ 172.8, 165.4, 138.8 (2C), 128.5 (2C), 127.8 (2C), 127.6 (2C), 99.0, 78.7, 78.5, 73.0 (2C), 72.9, 72.8, 70.5 (2C), 36.3, 36.2, 31.4, 31.2, 31.1, 30.0, 29.9, 29.8 (2C), 23.5, 23.4, 22.3; *m/z* **HRMS** (ES^+) $C_{35}H_{48}NO_5$: 562.3527 $[M+H]^+$, found 562.3520.

3-[(2S,6S)-6-[4-(benzyloxy)butyl]tetrahydropyranyl]-5-[(2R',6S')-6'-[4-(benzyloxy)butyl]tetrahydropyranyl]isoxazole 11

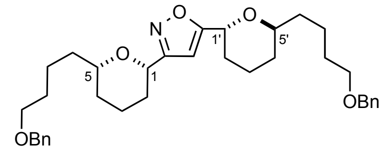

General procedure C was followed with *t*-BuOCl (14.6 µl, 134 µmol) added to a solution of oxime **3** (37.0 mg, 130 µmol) in CH_2Cl_2 (1 mL) followed by alkyne **8** (50.0 mg, 180 µmol) and Et_3N (26.0 µl, 190 µmol) in CH_2Cl_2 (1 mL). Purification by flash column

chromatography (5% EtOAc/hexane) provided isoxazole **11** (15.6 mg, 22%) as a colourless oil.

R$_f$ 0.37 (20% EtOAc/hexane); $[\alpha]_D^{20}$ -22.9 (*c* 1.5, $CHCl_3$); **IR** (thin film) 2955, 2909, 2839 cm^{-1}; **^{1}H NMR** (300 MHz, $CDCl_3$) δ 7.33-7.24 (10H, m, ArH), 6.22 (1H, s, ArH), 5.01 (1H, t, *J* = 4.2 Hz, $H_{1'}$), 4.55-4.51 (1H, m, H_1), 4.49 (4H, s, OCH_2Ar), 3.61-3.52 (1H, m, $H_{5'}$), 3.52-3.42 (1H, m, H_5), 3.47 (4H, t, *J* = 6.4 Hz, H_9 + $H_{9'}$), 2.06-1.88 (4H, m, H_{2A} + $H_{2A'}$ + H_{3A} + $H_{3A'}$), 1.76-0.79 (20H, m, H_{2B} + $H_{2B'}$ + H_{3B} + $H_{3B'}$ + H_4 + $H_{4'}$ + H_6 + $H_{6'}$ + H_7 + $H_{7'}$ + H_8 + $H_{8'}$); **^{13}C NMR** (75 MHz, $CDCl_3$) δ 172.8, 165.9, 139.0 (2C), 128.7 (2C), 128.0 (2C), 127.9 (2C), 100.9, 78.9, 77.2, 73.3, 73.2, 72.7, 70.7, 70.6, 68.7, 36.6, 35.4, 31.5, 31.4, 30.9, 30.2, 27.9, 27.7, 23.8, 22.6, 22.5, 19.7; *m/z* **HRMS** (ES^+) $C_{35}H_{48}NO_5$: 562.3527 $[M+H]^+$, found 562.3517.

3-[(2*R*,6*S*)-6-[4-(benzyloxy)butyl]tetrahydropyranyl]-5-[(2*S*',6*S*')-6'-[4-(benzyloxy)butyl]tetrahydropyranyl]isoxazole 12

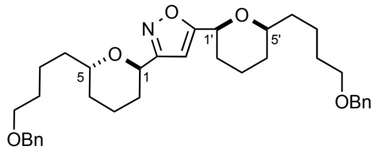

General procedure C was followed with *t*-BuOCl (14.6 µl, 134 µmol) added to a solution of oxime **4** (37.0 mg, 130 µmol) in CH_2Cl_2 (1 mL) followed by alkyne **7** (50.0 mg, 180 µmol) and Et_3N (26.0 µl, 190 µmol) in CH_2Cl_2 (1 mL). Purification by flash column chromatography (5% EtOAc/hexane) provided isoxazole **12** (16.0 mg, 22%) as a colourless oil.

R$_f$ 0.22 (10% EtOAc/hexane); $[\alpha]_D^{20}$ –32.8 (*c* 1.6, $CHCl_3$); **IR** (thin film) 3030, 2936, 2857, 2795, 1719, 1603 cm^{-1}; **^{1}H NMR** (400 MHz, $CDCl_3$) δ 7.36-7.25 (10H, m, ArH), 6.20 (1H, s, ArH), 4.95 (1H, dd, *J* = 4.7, 4.0 Hz, $H_{1'}$), 4.52-4.48 (1H, m, H_1), 4.49 (4H, s, OCH_2Ar), 3.52-3.42 (2H, m, H_5 + $H_{5'}$), 3.47 (4H, t, *J* = 6.5 Hz, H_9 + $H_{9'}$), 2.17-2.11 (1H, dq, J 12.5, 4.0 Hz, $H_{2A'}$), 1.96-1.86 (3H, m, H_{2A} + H_{3A} + $H_{3A'}$), 1.74-1.26 (20H, m, H_{2B} + $H_{2B'}$ + H_{3B} + $H_{3B'}$ + H_4 + $H_{4'}$ + H_6 + $H_{6'}$ + H_7 + $H_{7'}$ + H_8 + $H_{8'}$); **^{13}C NMR** (100 MHz, $CDCl_3$) δ 173.3, 164.7, 138.8 (2C), 129.7 (2C), 128.5, 127.8, 127.7, 127.6, 99.9, 78.7, 73.0, 72.8, 72.3, 70.5, 70.4, 69.0, 68.6, 36.3, 34.9, 31.1, 30.7, 30.4, 29.9 (2C), 27.7, 23.4, 22.3, 22.2, 19.3; *m/z* **HRMS** ($ASAP^+$) Calc. for $C_{35}H_{51}O_5N_2$ $[M+NH_4]^+$ 579.3792, found 579.3779.

3,5-bis(2R,6S)-5-[4-(benzyloxy)butyl]tetrahydropyranyl)isoxazole 13

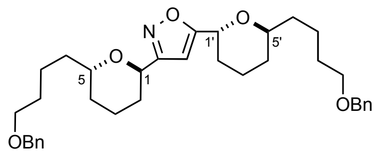

General procedure C was followed with *t*-BuOCl (11.9 µl, 109 µmol) added to a solution of oxime **4** (30.0 mg, 100 µmol) in CH_2Cl_2 (1 mL) followed by alkyne **8** (45.6 mg, 170 µmol) and Et_3N (21.1 µl, 154 µmol) in CH_2Cl_2 (1 mL). Purification by flash column chromatography (5% EtOAc/hexane) provided isoxazole **13** (11.5 mg, 20%) as a colourless oil.

$[\alpha]_D^{20}$ –20.6 (*c* 1.4, $CHCl_3$); **IR** (thin film) 2932, 2855, 2785 cm^{-1}; **^{1}H NMR** (300 MHz, $CDCl_3$) δ 7.35-7.25 (10H, m, Ar$\underline{H}$), 6.20 (1H, s, Ar$\underline{H}$), 5.04 (1H, dd, *J* = 5.4, 3.4 Hz, $\underline{H_{1'}}$), 4.98 (1H, dd, *J* = 5.1, 3.4 Hz, $\underline{H_1}$), 4.50 (2H, s, OC$\underline{H_2}$Ar), 4.49 (2H, s, OC$\underline{H_2}$Ar), 3.59-3.54 (1H, m, $\underline{H_{5'}}$), 3.51-3.43 (1H, m, $\underline{H_5}$), 3.47 (2H, t, *J* = 6.6 Hz, $\underline{H_{9'}}$), 3.46 (2H, t, *J* = 6.5 Hz, $\underline{H_9}$), 2.17-2.14 (1H, m, $\underline{H_{2A}}$), 2.07-2.03 (1H, m, $\underline{H_{2A'}}$), 1.98-1.88 (2H, m, $\underline{H_3}$), 1.75-1.28 (20H, m, $\underline{H_{2B}}$ + $\underline{H_{2B'}}$ + $\underline{H_{3B}}$ + $\underline{H_{3B'}}$ + $\underline{H_4}$ + $\underline{H_{4'}}$ + $\underline{H_6}$ + $\underline{H_{6'}}$ + $\underline{H_7}$ + $\underline{H_{7'}}$ + $\underline{H_8}$ + $\underline{H_{8'}}$); **^{13}C NMR** (75 MHz, $CDCl_3$) δ 173.2, 165.1, 139.0 (2C), 128.7 (2C), 128.1 (2C), 128.0, 127.9, 101.8, 73.3 (2C), 72.8, 72.7, 70.7 (2C), 68.9, 68.8, 35.5, 35.3, 31.0, 30.9, 30.2 (2C), 27.9, 27.8, 22.6, 22.5, 19.7, 19.6; *m/z* **HRMS** (ES^+) $C_{35}H_{48}NO_5$: 562.3527 $[M+H]^+$, found 562.3520.

3,4-(bis(2*S*,6*S*)-6-[4-(benzyloxy)butyl]tetrahydropyranyl)furoxan 14

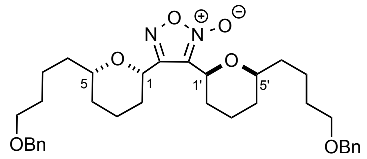

General procedure C was followed with *t*-BuOCl (13.0 µl, 119 µmol) added to a solution of oxime **3** (33.0 mg, 113 µmol) in CH_2Cl_2 (1 mL) followed by Et_3N (24.0 µl, 167 µmol). Purification by flash column chromatography (10% EtOAc/hexane) provided furoxan **14** (14.5 mg, 44%) as a colourless oil.

R_f 0.22 (10% EtOAc/hexane); $[\alpha]_D^{20}$ –25.0 (*c* 0.6, $CHCl_3$); **IR** (ATR) 2936, 2859, 1736, 1719, 1607 cm^{-1}; **^{1}H NMR** (400 MHz, $CDCl_3$) δ 7.35-7.25 (10H, m, Ar$\underline{H}$), 4.64-4.60 (1H, m, $\underline{H_{1'}}$), 4.57 (1H, dd, *J* = 11.4, 2.5 Hz, $\underline{H_1}$), 4.49 (2H, s, OC$\underline{H_2}$Ar), 4.48 (2H, s, OC$\underline{H_2}$Ar), 3.46 (4H, d, *J* = 6.5, Hz, $\underline{H_9}$ + $\underline{H_{9'}}$), 3.44-3.37 (2H, m, $\underline{H_5}$ + $\underline{H_{5'}}$), 1.97-1.86 (4H, m, $\underline{H_{2A}}$ + $\underline{H_{2A'}}$ + $\underline{H_{3A}}$ + $\underline{H_{3A'}}$), 1.78-1.74 (1H, m, $\underline{H_{4A'}}$), 1.73-1.27 (19H, m, $\underline{H_{2B}}$ + $\underline{H_{2B'}}$ + $\underline{H_{3B}}$ + $\underline{H_{3B'}}$ + $\underline{H_{4A}}$ + $\underline{H_{4B}}$ + $\underline{H_{4B'}}$ + $\underline{H_{6\text{-}8}}$ + $\underline{H_{6'\text{-}8'}}$); **^{13}C NMR** (100 MHz, $CDCl_3$) δ 158.0, 138.7 (2C), 128.5

(2C), 127.7 (2C), 127.6 (2C), 115.5, 79.1 (2C), 73.1 (2C), 72.7, 71.3, 70.4 (2C), 36.4, 36.3, 31.1, 31.0, 29.9 (2C), 28.9, 26.9, 23.3, 23.2, 22.6, 22.4; *m/z* **HRMS** (ES^+) Calc. for $C_{34}H_{47}O_6N_2$ $[M+H]^+$ 579.3429, found 579.3428.

3,4-(bis(2*R*,6*S*)-6-[4-(benzyloxy)butyl]tetrahydropyranyl)furoxan 15

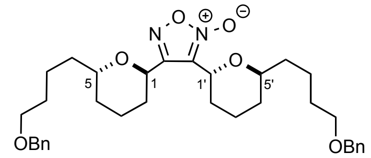

General procedure C was followed with *t*-BuOCl (27.5 µl, 252 µmol) added to a solution of oxime **4** (70.0 mg, 240 µmol) in CH_2Cl_2 (2 mL) followed by Et_3N (49.0 µl, 360 µmol). Purification by flash column chromatography (10% EtOAc/hexane) provided furoxan **15** (42.0 mg, 61%) as a colourless oil.

R$_f$ 0.28 (10% EtOAc/hexane); $[\alpha]_D^{20}$ –15.0 (*c* 1.3, $CHCl_3$); **IR** (thin film) 2934, 2849, 1670, 1599, 1560, 1521 cm^{-1}; **^{1}H NMR** (400 MHz, $CDCl_3$) δ 7.36-7.27 (10H, m, Ar$\underline{H}$), 5.12 (1H, dd, *J* = 4.9, 3.0 Hz, $\underline{H_{1'}}$), 5.04 (1H, m, $\underline{H_1}$), 4.49 (2H, s, OC$\underline{H_2}$Ar), 4.48 (2H, s, OC$\underline{H_2}$Ar), 3.49-3.42 (1H, m, $\underline{H_{5'}}$), 3.44 (2H, d, *J* = 6.4, Hz, $\underline{H_{9'}}$), 3.43 (2H, d, *J* = 6.4, Hz, $\underline{H_9}$), 3.39-3.35 (1H, m, $\underline{H_5}$), 2.22-2.14 (2H, m, $\underline{H_{2A}}$ + $\underline{H_{2A'}}$), 1.98-1.84 (2H, m, $\underline{H_{3A}}$ + $\underline{H_{3A'}}$), 1.73-1.27 (20H, m, $\underline{H_{2B}}$ + $\underline{H_{2B'}}$ + $\underline{H_{3B}}$ + $\underline{H_{3B'}}$ + $\underline{H_4}$ + $\underline{H_{4'}}$ + $\underline{H_{6-8}}$ + $\underline{H_{6'-8'}}$); **^{13}C NMR** (100 MHz, $CDCl_3$) δ 157.8, 138.7 (2C), 128.5 (2C), 127.8 (2C), 127.7 (2C), 114.5, 73.6, 73.1 (2C), 72.8, 70.4 (2C), 67.1, 64.8, 35.5, 33.4, 30.7, 30.0 (2C), 29.8, 29.4, 25.7, 22.6, 22.5, 19.5, 19.1; *m/z* **HRMS** ($ASAP^+$) Calc. for $C_{34}H_{47}O_6N_2$ $[M+H]^+$ 579.3429, found 579.3423.

3,4-(bis(2*R*,6*S*)-6-hexyltetrahydropyranyl)furoxan 16

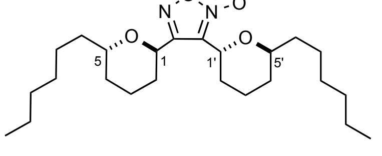

General procedure C was followed with *t*-BuOCl (26.0 µl, 221 µmol) added to a solution of oxime **5** (45.0 mg, 211 µmol) in CH_2Cl_2 (2 mL) followed by Et_3N (43.0 µl, 317 µmol). Purification by flash column chromatography (5% Et_2O/hexane) provided furoxan **16** (28.0 mg, 64%) as a colourless oil.

R$_f$ 0.29 (5% Et_2O/hexane); $[\alpha]_D^{20}$ –20.0 (*c* 0.9, $CHCl_3$); **IR** (thin film) 2928, 2857, 1601, 1506 cm^{-1}; **^{1}H NMR** (400 MHz, $CDCl_3$) δ 5.14 (1H, dd, *J* = 4.9, 2.8 Hz, $\underline{H_{1'}}$), 5.06 (1H, dd, *J* = 4.8, 4.0 Hz, $\underline{H_1}$), 3.49-3.43 (1H, m, $\underline{H_{5'}}$), 3.39-3.34 (1H, m, $\underline{H_5}$), 2.22-2.15 (2H, m, $\underline{H_{2A}}$

+ $\underline{H}_{2A'}$), 1.98-1.89 (2H, m, $\underline{H}_{3A}$ + $\underline{H}_{3A'}$), 1.73-1.27 (28H, m, $\underline{H}_{2B}$ + $\underline{H}_{2B'}$ + $\underline{H}_{3B}$ + $\underline{H}_{3B'}$ + $\underline{H}_{4}$ + $\underline{H}_{4'}$ + $\underline{H}_{6\text{-}10}$ + $\underline{H}_{6'\text{-}10'}$), 0.87 (3H, t, *J* = 6.8 Hz, $\underline{H}_{11'}$), 0.86 (3H, t, *J* = 6.7 Hz, $\underline{H}_{11}$); **^{13}C NMR** (100 MHz, $CDCl_3$) δ 157.9, 114.6, 73.7, 72.9, 67.0, 64.8, 35.6, 33.5, 31.9 (2 x), 30.8, 29.5 (2 x), 29.3, 25.9, 25.7, 25.7, 25.0, 22.7 (2 x), 19.5, 19.2, 14.2 (2 x); *m/z* **HRMS** ($ASAP^+$) Calc. for $C_{24}H_{43}O_4N_2$ $[M+H]^+$ 423.3323, found 423.3216.

3,4-(bis(2*S*,6*S*)-6-[4-(benzyloxy)butyl]tetrahydropyranyl)furazan 17

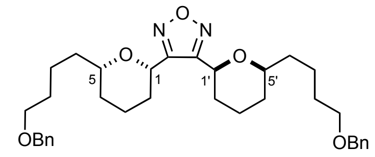

To a solution of furoxan **11** (14.5 mg, 25.0 μmol) in methanol (1.5 mL) was added zinc dust (3.60 mg, 55.0 μmol) and ammonium formate (5.20 mg, 82.0 μmol) and the resulting mixture heated to reflux overnight. The mixture was then filtered through a plug of Celite, the cake washed with methanol and the combined filtrate concentrated in vacuo. Purification by flash column chromatography (10% EtOAc/hexane) provided furazan **14** (3.1 mg, 22%) as a colourless oil.

R_f 0.61 (20% EtOAc/hexane); $[\alpha]_D^{20}$ +6.7 (*c* 0.3, $CHCl_3$); **IR** (ATR) 2936, 2855, 1719 cm^{-1}; **^{1}H NMR** (500 MHz, $CDCl_3$) δ 7.34-7.27 (10H, m, Ar$\underline{H}$), 4.70 (2H, dd, *J* = 11.3, 2.2 Hz, $\underline{H}_1$), 4.49 (4H, s, OC$\underline{H}_2$Ar), 3.49-3.46 (2H, m, $\underline{H}_5$), 3.47 (4H, t, *J* = 6.4 Hz, $\underline{H}_9$), 2.07-2.04 (2H, m, $\underline{H}_{2A}$), 1.96-1.85 (4H, m, $\underline{H}_{2B}$ + $\underline{H}_{3A}$), 1.66-1.25 (18H, m, $\underline{H}_{3B}$ + $\underline{H}_4$ + $\underline{H}_6$ + $\underline{H}_7$ + $\underline{H}_8$); **^{13}C NMR** (125 MHz, $CDCl_3$) δ 154.8 (2C), 138.7 (2C), 128.5 (2C), 127.7 (4C), 79.1 (2C), 73.1 (2C), 71.3 (2C), 70.4 (2C), 36.4 (2C), 31.3 (2C), 30.0 (2C), 29.2 (2C), 23.4 (2C), 22.9 (2C); *m/z* **HRMS** (ES^+) Calc. for $C_{34}H_{47}O_5N_2$ $[M+H]^+$ 563.3479, found 563.3477.

3,4-(bis(2*R*,6*S*)-6-[4-(benzyloxy)butyl]tetrahydropyranyl)furazan 18

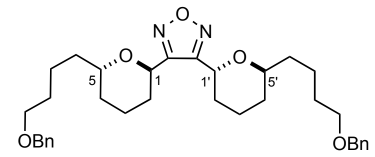

Furoxan **15** (11.0 mg, 19.0 μmol) was dissolved in triethyl phosphite (0.3 mL) and heated to reflux overnight. The mixture was directly purified by flash column chromatography (10% EtOAc/hexane) to provide furazan **18** (8.0 mg, 77%) as a colourless oil.

R_f 0.45 (15% EtOAc/hexane); $[\alpha]_D^{20}$ –47.5 (*c* 0.8, $CHCl_3$); **IR** (ATR) 2936, 2855, 1717 cm^{-1}; **^{1}H NMR** (400 MHz, $CDCl_3$) δ 7.36-7.25 (10H, m, Ar$\underline{H}$), 5.23 (2H, dd, *J* = 4.7, 2.7 Hz,

H1), 4.47 (4H, s, OCH2Ar), 3.42 (4H, t, J = 6.5 Hz, H9), 3.20 (2H, dtt, J = 9.5, 4.7, 2.3 Hz, H5), 2.17-2.13 (2H, m, H2A), 2.03-1.91 (4H, m, H2B + H3A), 1.77-1.25 (18H, m, H3B + H4 + H6 + H7 + H8); **^{13}C NMR** (100 MHz, $CDCl_3$) δ 154.2 (2C), 138.7 (2C), 128.5 (2C), 127.8 (2C), 127.6 (2C), 73.1 (2C), 72.3 (2C), 70.3 (2C), 65.9 (2C), 35.5 (2C), 30.9 (2C), 29.9 (2C), 26.8 (2C), 22.4 (2C), 19.1 (2C); *m/z* **HRMS** (ASAP$^+$) Calc. for $C_{34}H_{47}O_5N_2$ $[M+H]^+$ 563.3479, found 563.3485.

3,4-(bis(2*R*,6*S*)-6-hexyltetrahydropyranyl)furazan 19

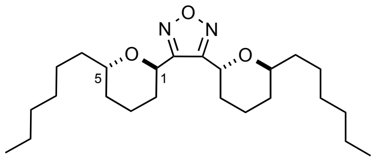

Furoxan **16** (19.0 mg, 45.0 μmol) was dissolved in triethyl phosphite (0.3 mL) and heated to reflux overnight. The mixture was directly purified by flash column chromatography (2% Et_2O/hexane) followed by a second column using the same solvent system to provide furazan **19** (10.7 mg, 59%) as a colourless oil.

R_f 0.15 (2% Et_2O/hexane); $[\alpha]_D^{20}$ –70.7 (*c* 1.1, $CHCl_3$); **IR** (ATR) 2926, 2855, 1458, 1441 cm^{-1}; **^{1}H NMR** (500 MHz, $CDCl_3$) δ 5.28 (2H, dd, J = 4.7, 2.6 Hz, H1), 322 (2H, dtt, J = 10.0, 4.8, 2.6 Hz, H5), 3.39-3.34 (2H, m, H5), 2.21-2.17 (2H, m, H3A), 2.09-1.98 (4H, m, H2A + H4A), 1.82-1.77 (2H, m, H2B), 1.67-1.52 (4H, m, H3B + H4B), 1.38-1.17 (20H, m, H6-10), 0.87 (3H, t, J = 6.8 Hz, H11); **^{13}C NMR** (125 MHz, $CDCl_3$) δ 154.2, 72.4, 65.9, 35.6, 31.9, 31.0, 29.3, 26.8, 25.7, 22.7, 19.2, 14.2; *m/z* **HRMS** (ES$^+$) Calc. for $C_{34}H_{42}O_3N_2$ $[M+H]^+$ 429.3088, found 429.3078.

References

[1] a) G. J. Florence, A. L. Fraser, E. R. Gould, E. F. King, S. K. Menzies, J. C. Morris, L. B. Tulloch, T. K. Smith, *ChemMedChem* **2014**, *9*, 2548 – 2556.

^{1}H and ^{13}C NMR Spectra for Selected Compounds

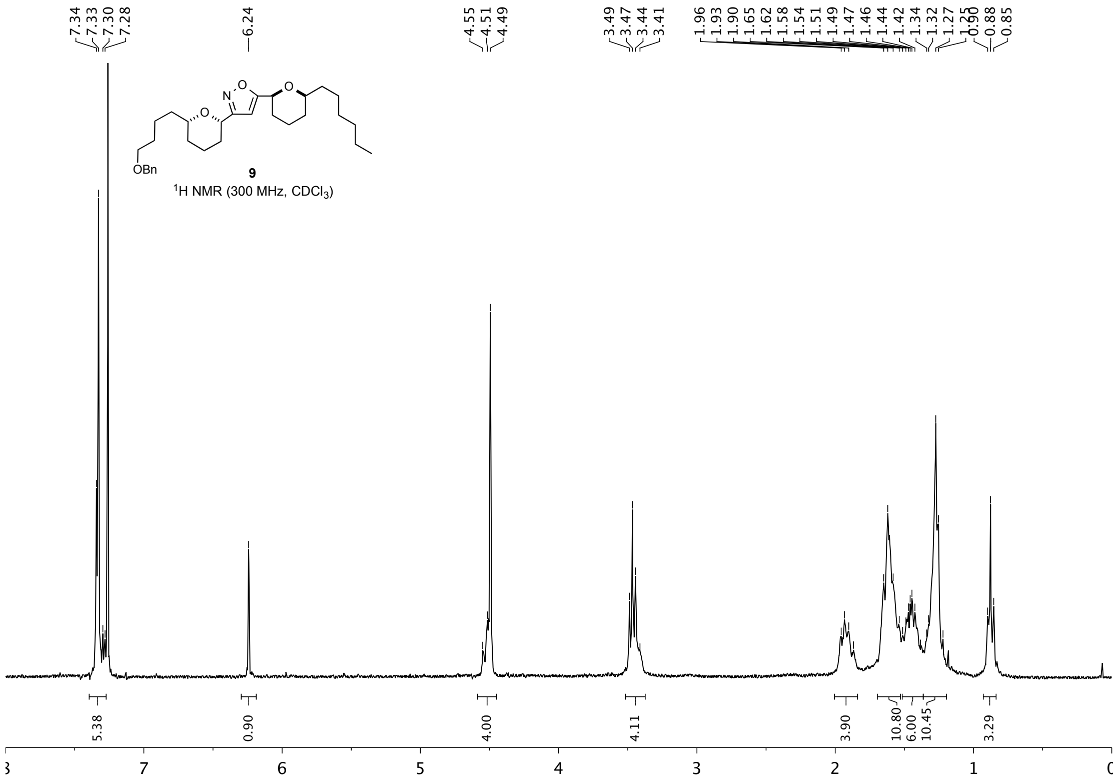
7.34
7.33
7.30
7.28
6.24
4.55
4.51
4.49
3.49
3.47
3.44
3.41
1.96
1.93
1.90
1.65
1.62
1.58
1.54
1.51
1.49
1.47
1.46
1.44
1.42
1.34
1.32
1.27
1.25
0.90
0.88
0.85
OBn
9
1H NMR (300 MHz, CDCl3)
5.38
0.90
4.00
4.11
3.90
10.80
6.00
10.45
3.29
7
6
5
4
3
2
1

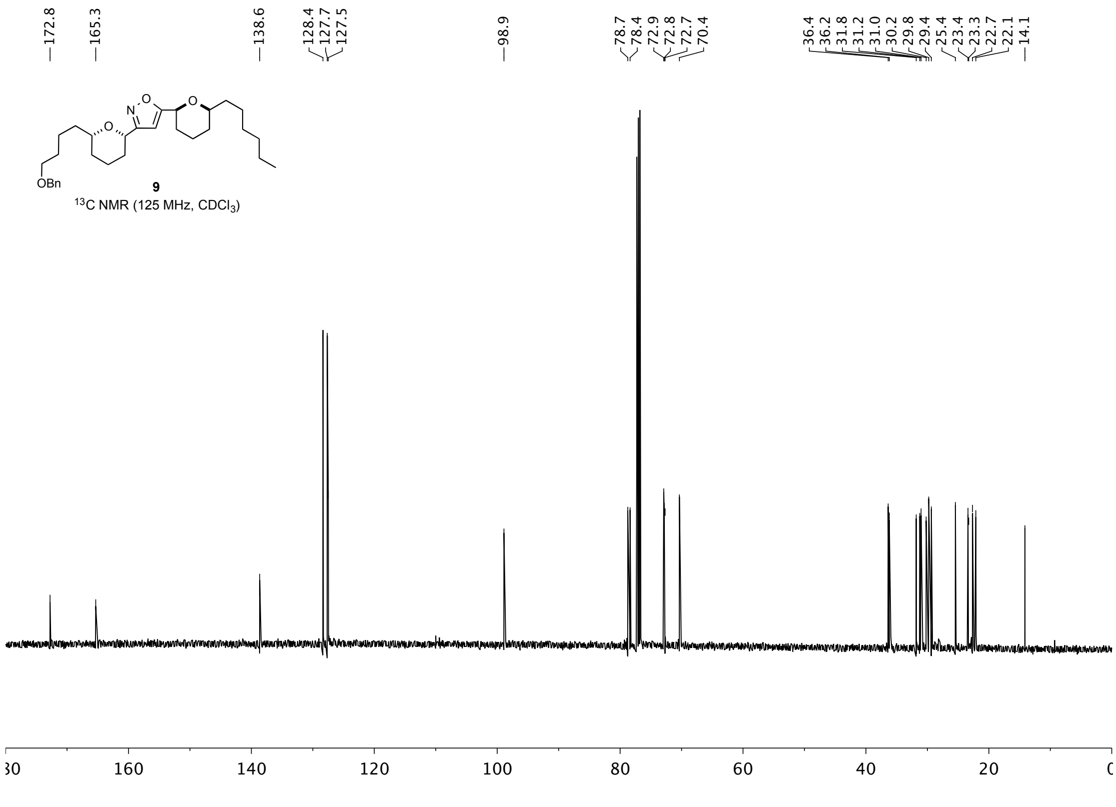
172.8
165.3
138.6
128.4
127.7
127.5
98.9
78.7
78.4
72.9
72.8
72.7
70.4
36.4
36.2
31.8
31.2
31.0
30.2
29.8
29.4
25.4
23.4
23.3
22.7
22.1
14.1
OBn
9
13C NMR (125 MHz, CDCl3)
160
140
120
100
80
60
40
20

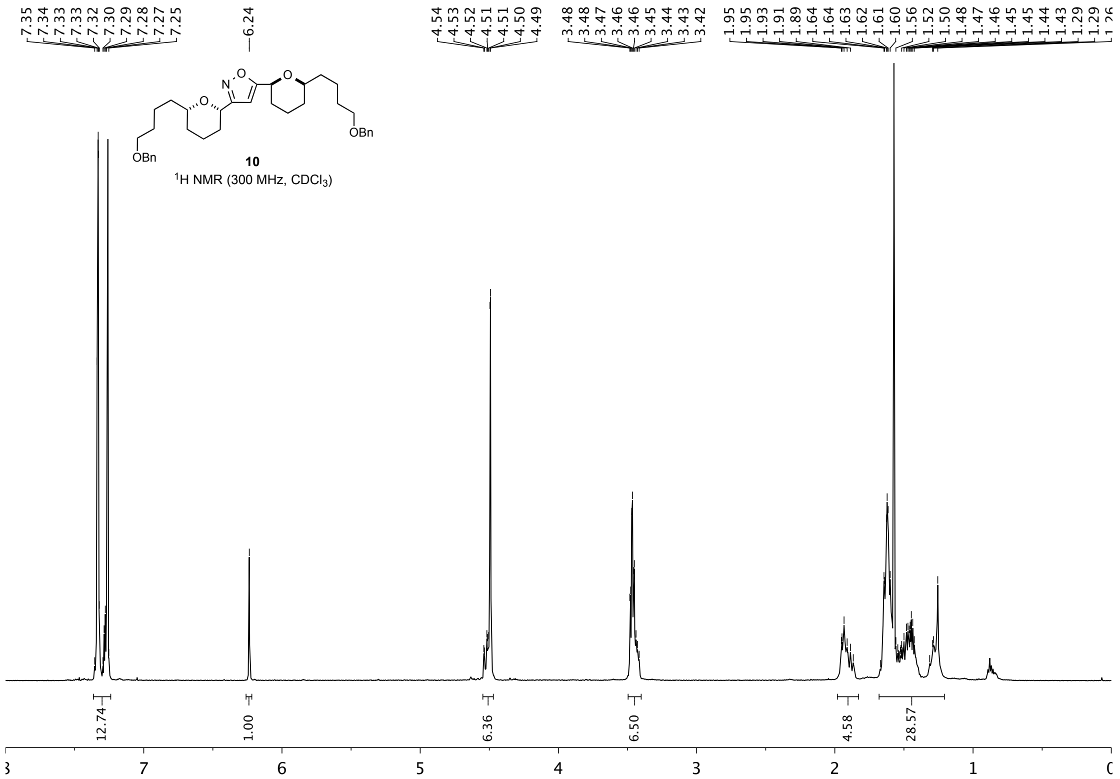

7.35 7.34 7.33 7.33 7.32 7.30 7.29 7.28 7.27 7.25
6.24
4.54 4.53 4.52 4.51 4.51 4.50 4.49
3.48 3.48 3.47 3.46 3.46 3.45 3.44 3.43 3.42
1.95 1.95 1.93 1.91 1.89 1.64 1.64 1.63 1.62 1.61 1.60 1.56 1.52 1.50 1.48 1.47 1.46 1.45 1.45 1.44 1.43 1.29 1.29
N-O
O
OBn
OBn
10
1H NMR (300 MHz, CDCl3)
12.74
1.00
6.36
6.50
4.58
28.57
7
6
5
4
3
2
1

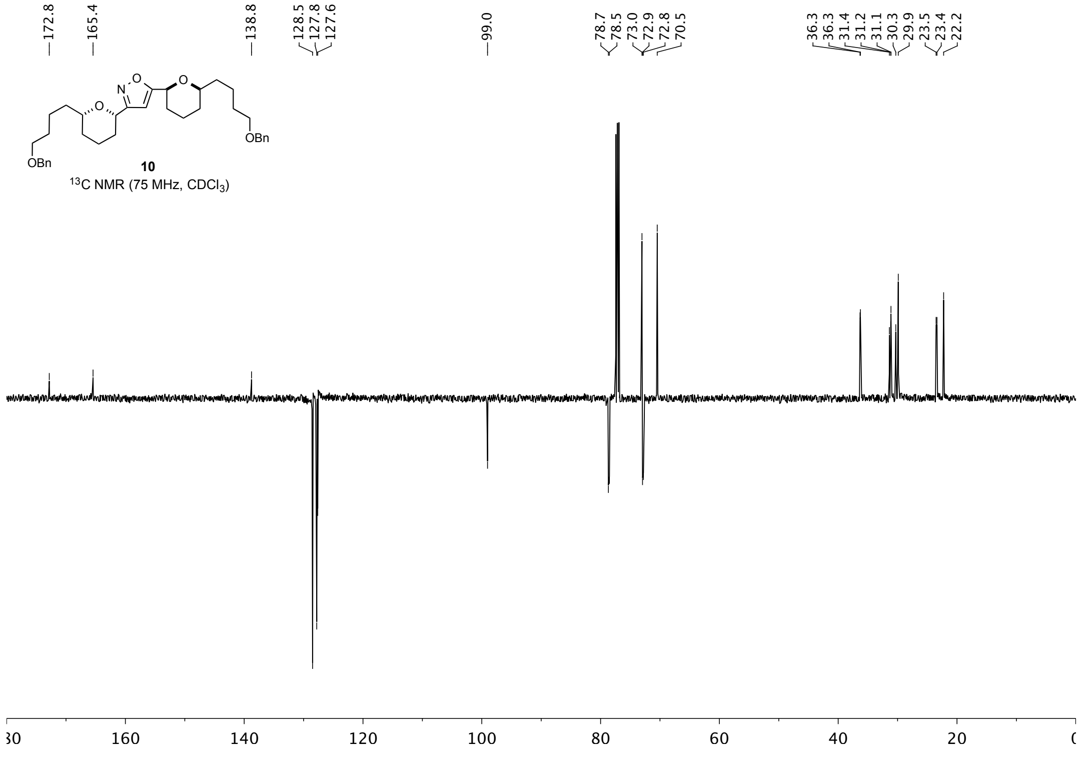

172.8
165.4
138.8
128.5 127.8 127.6
99.0
78.7 78.5 73.0 72.9 72.8 70.5
36.3 36.3 31.4 31.2 31.1 30.3 29.9 23.5 23.4 22.2
N-O
O
OBn
OBn
10
13C NMR (75 MHz, CDCl3)
160
140
120
100
80
60
40
20

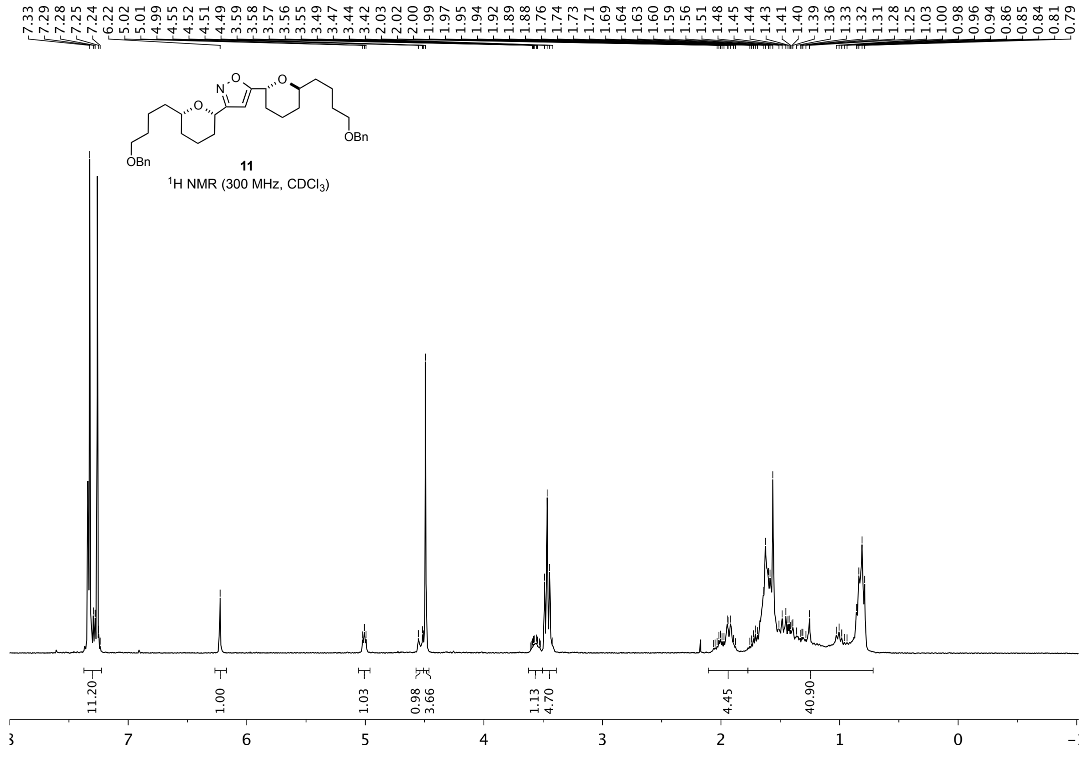

11

^{1}H NMR (300 MHz, CDCl$_3$)

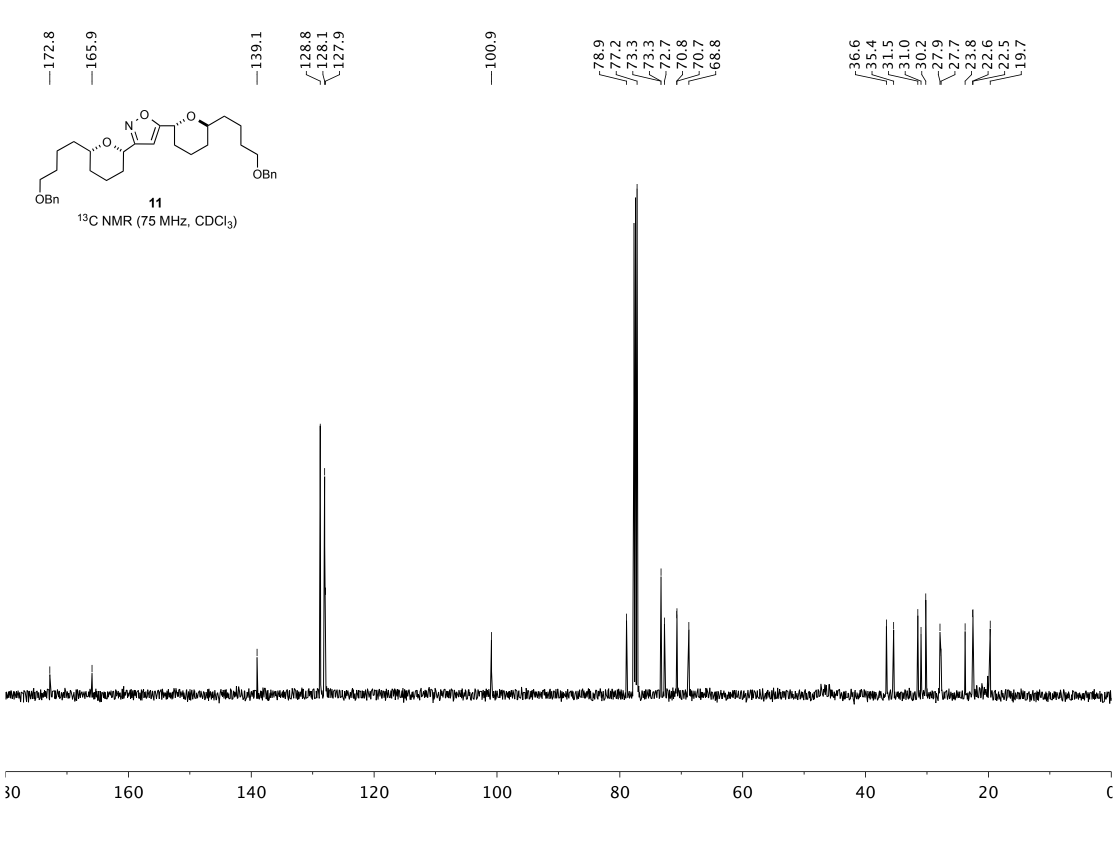

11

^{13}C NMR (75 MHz, CDCl$_3$)

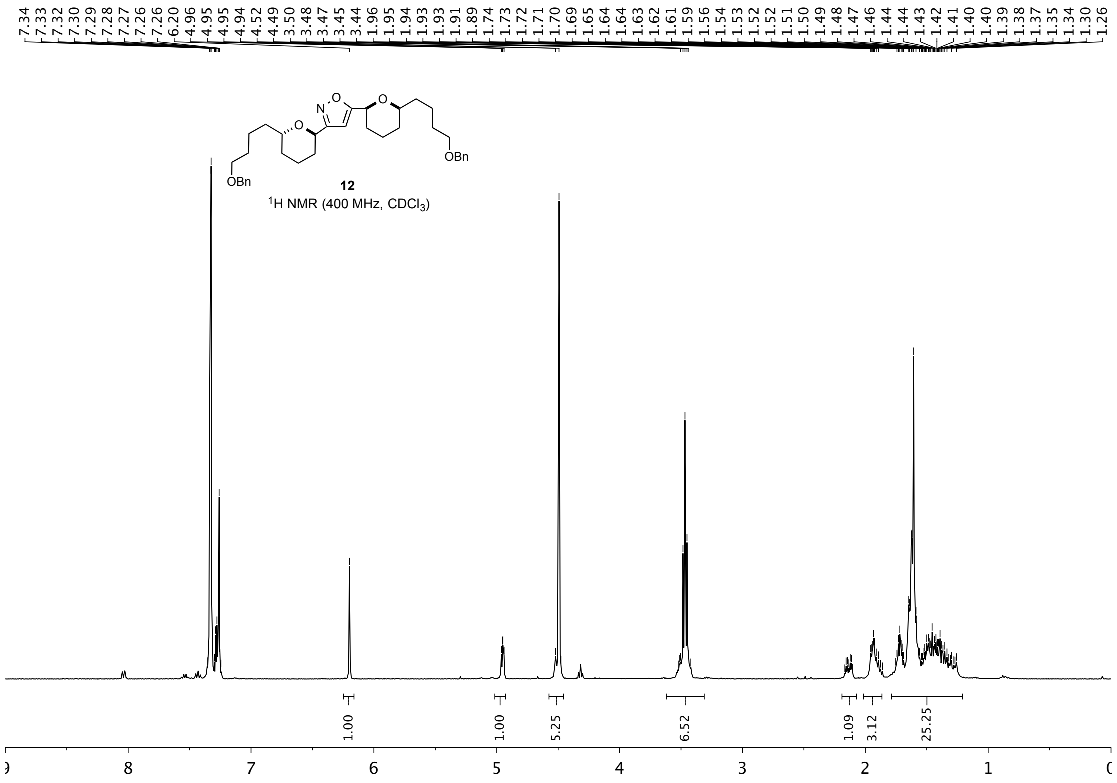

12
^{1}H NMR (400 MHz, $CDCl_3$)

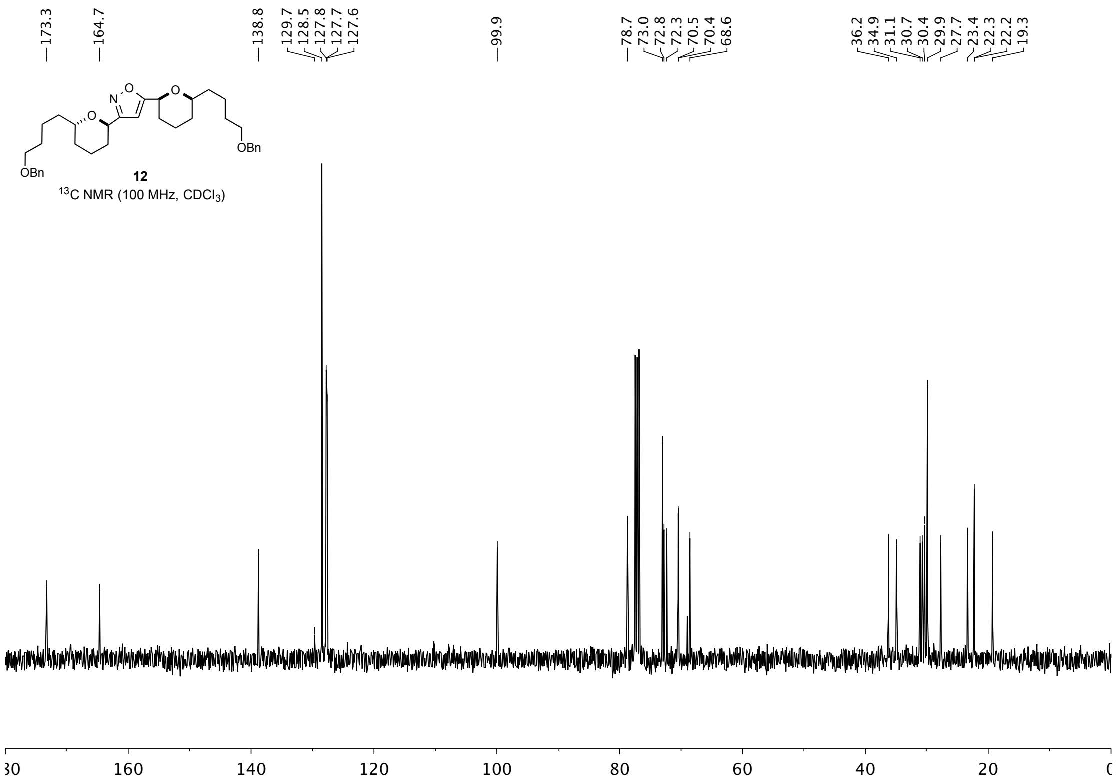

12
^{13}C NMR (100 MHz, $CDCl_3$)

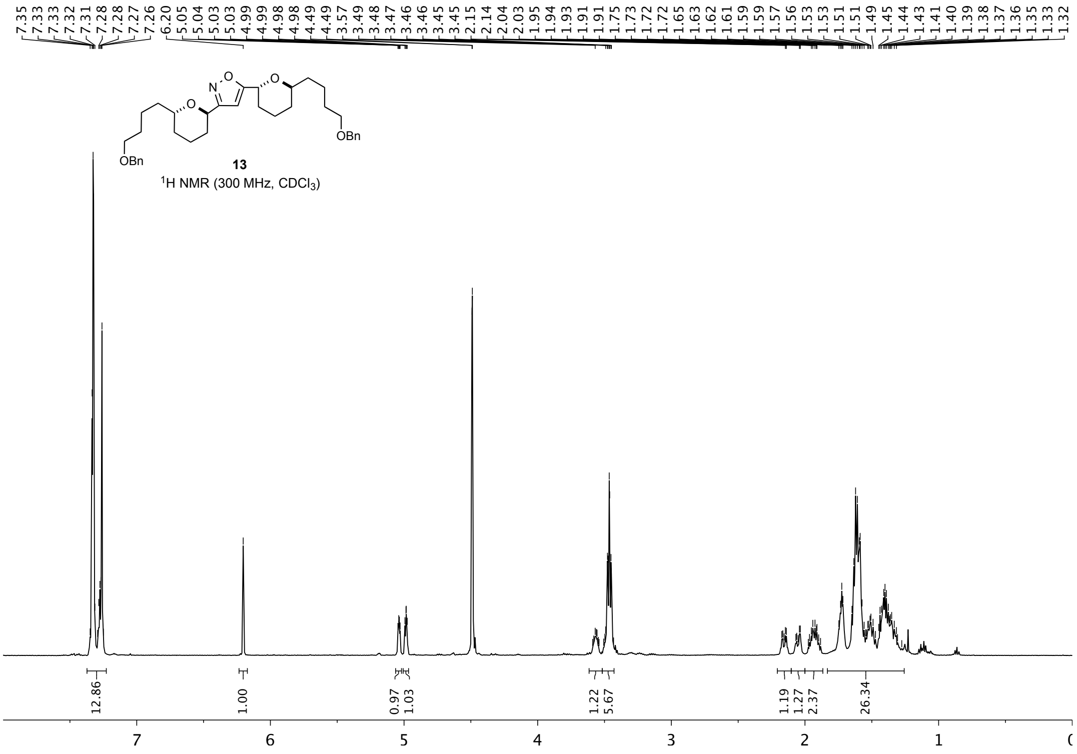
7.35 7.33 7.32 7.31 7.28 7.27 7.26 6.20 5.05 5.04 5.03 5.03 4.99 4.98 4.98 4.49 4.49 3.57 3.49 3.48 3.47 3.46 3.46 3.45 2.15 2.14 2.04 2.03 1.95 1.94 1.93 1.91 1.91 1.75 1.73 1.72 1.72 1.65 1.63 1.62 1.61 1.59 1.59 1.57 1.56 1.53 1.53 1.51 1.51 1.49 1.45 1.44 1.43 1.41 1.40 1.39 1.38 1.37 1.36 1.35 1.33 1.32
OBn
OBn
13
1H NMR (300 MHz, CDCl3)
12.86
1.00
0.97
1.03
1.22
5.67
1.19
1.27
2.37
26.34

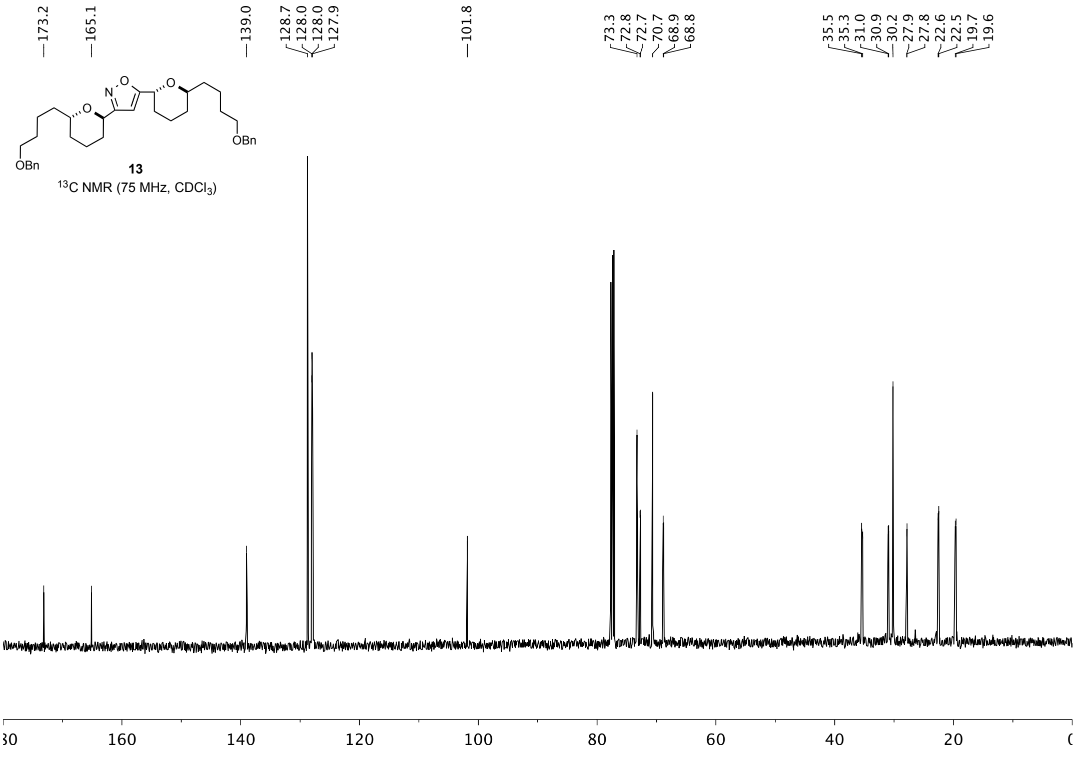
173.2
165.1
139.0
128.7 128.0 128.0 127.9
101.8
73.3 72.8 72.7 70.7 68.9 68.8
35.5 35.3 31.0 30.9 30.2 27.9 27.8 22.6 22.5 19.7 19.6
OBn
OBn
13
13C NMR (75 MHz, CDCl3)

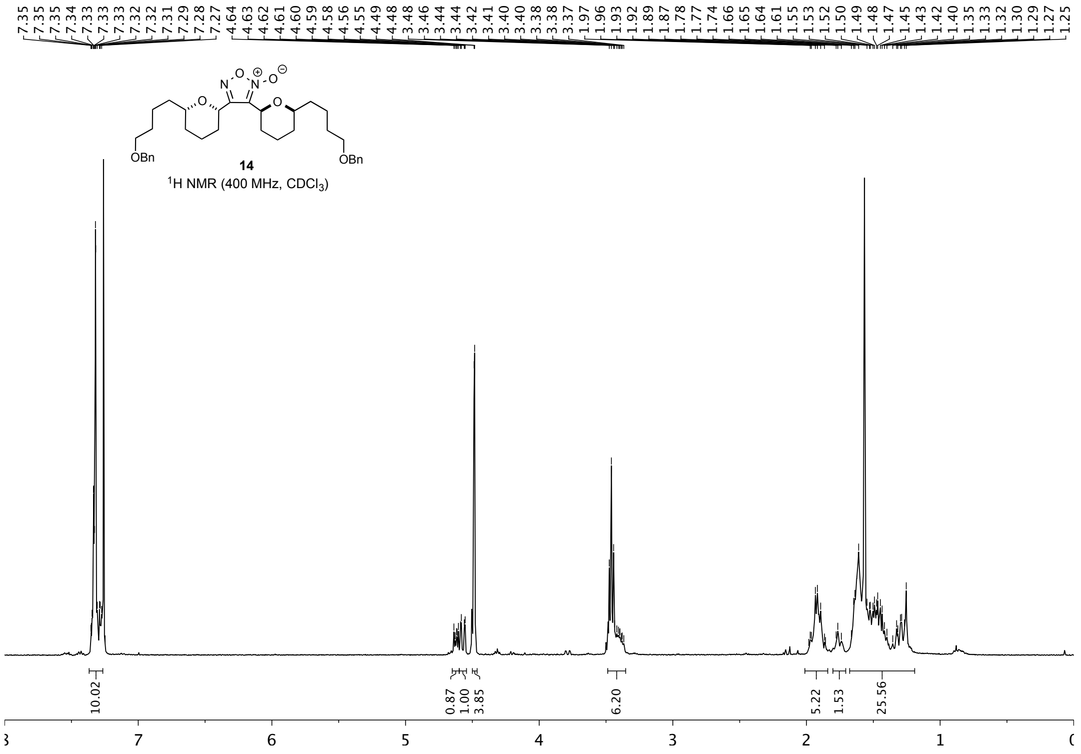
7.35 7.35 7.34 7.33 7.33 7.33 7.32 7.32 7.31 7.29 7.28 7.27 4.64 4.63 4.62 4.61 4.60 4.59 4.58 4.56 4.55 4.49 4.48 3.48 3.46 3.44 3.44 3.42 3.41 3.40 3.40 3.38 3.38 3.37 1.97 1.96 1.93 1.92 1.89 1.87 1.78 1.77 1.74 1.66 1.65 1.64 1.61 1.55 1.53 1.52 1.50 1.49 1.48 1.47 1.45 1.43 1.42 1.40 1.35 1.33 1.32 1.30 1.29 1.27 1.25
OBn
OBn
14
^{1}H NMR (400 MHz, CDCl$_3$)
10.02
0.87
1.00
3.85
6.20
5.22
1.53
25.56
8 7 6 5 4 3 2 1 0

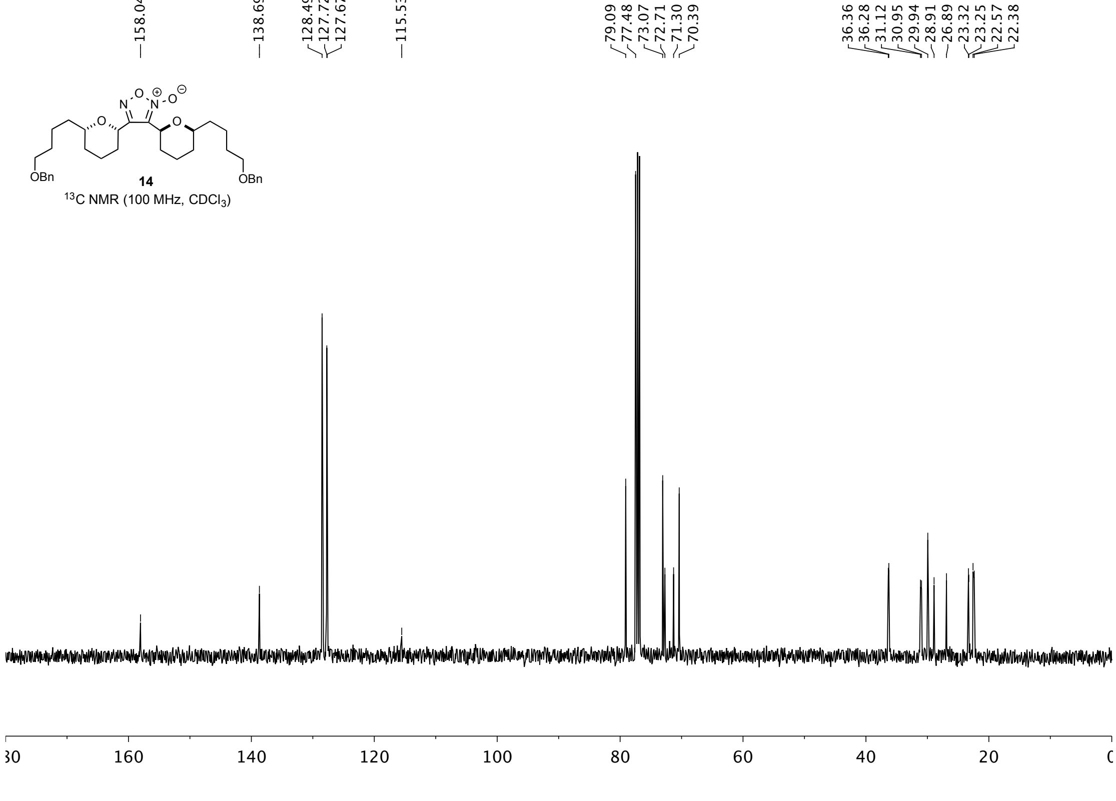
158.04
138.69
128.49 127.72 127.67
115.53
79.09 77.48 73.07 72.71 71.30 70.39
36.36 36.28 31.12 30.95 29.94 28.91 26.89 23.32 23.25 22.57 22.38
OBn
OBn
14
^{13}C NMR (100 MHz, CDCl$_3$)
180 160 140 120 100 80 60 40 20 0

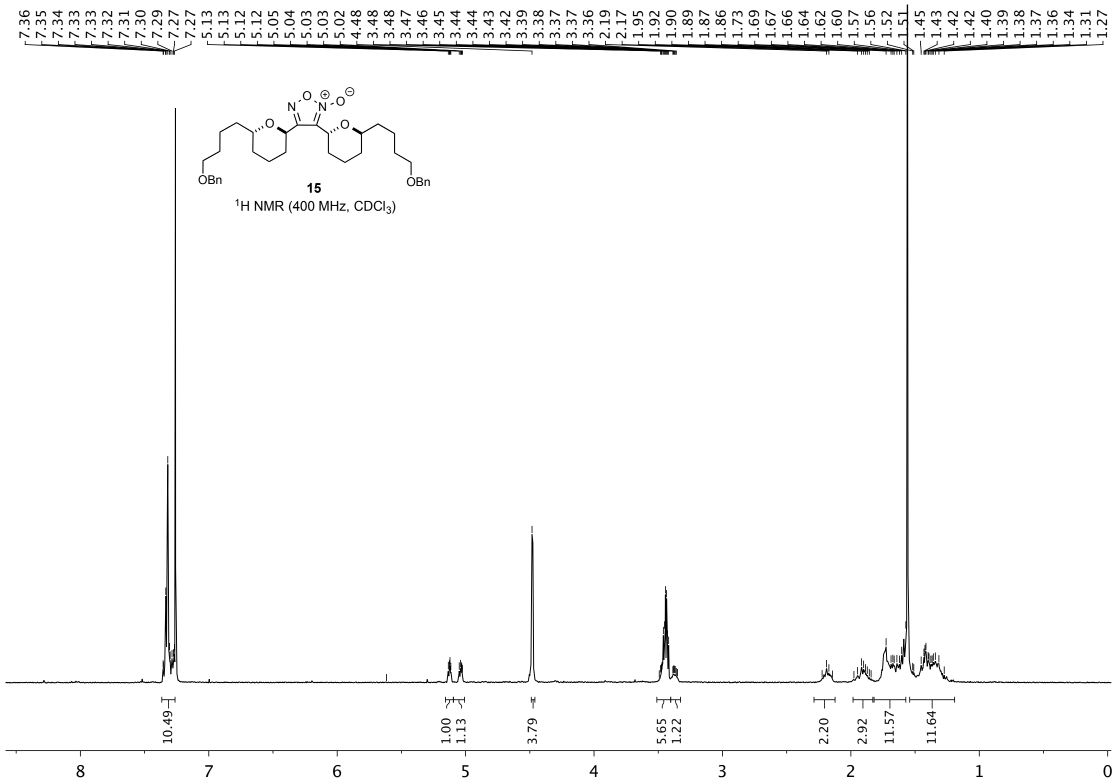
OBn
OBn
15
1H NMR (400 MHz, CDCl3)
7.36 7.35 7.34 7.33 7.33 7.32 7.31 7.30 7.29 7.27 7.27 5.13 5.13 5.12 5.12 5.05 5.04 5.03 5.03 5.02 4.48 3.48 3.48 3.47 3.46 3.45 3.44 3.44 3.43 3.42 3.39 3.38 3.37 3.37 3.36 2.19 2.17 1.95 1.92 1.90 1.89 1.87 1.86 1.73 1.69 1.67 1.66 1.64 1.62 1.62 1.60 1.57 1.56 1.52 1.51 1.45 1.43 1.42 1.42 1.40 1.39 1.38 1.37 1.36 1.34 1.31 1.27
10.49 1.00 1.13 3.79 5.65 1.22 2.20 2.92 11.57 11.64
8 7 6 5 4 3 2 1 0

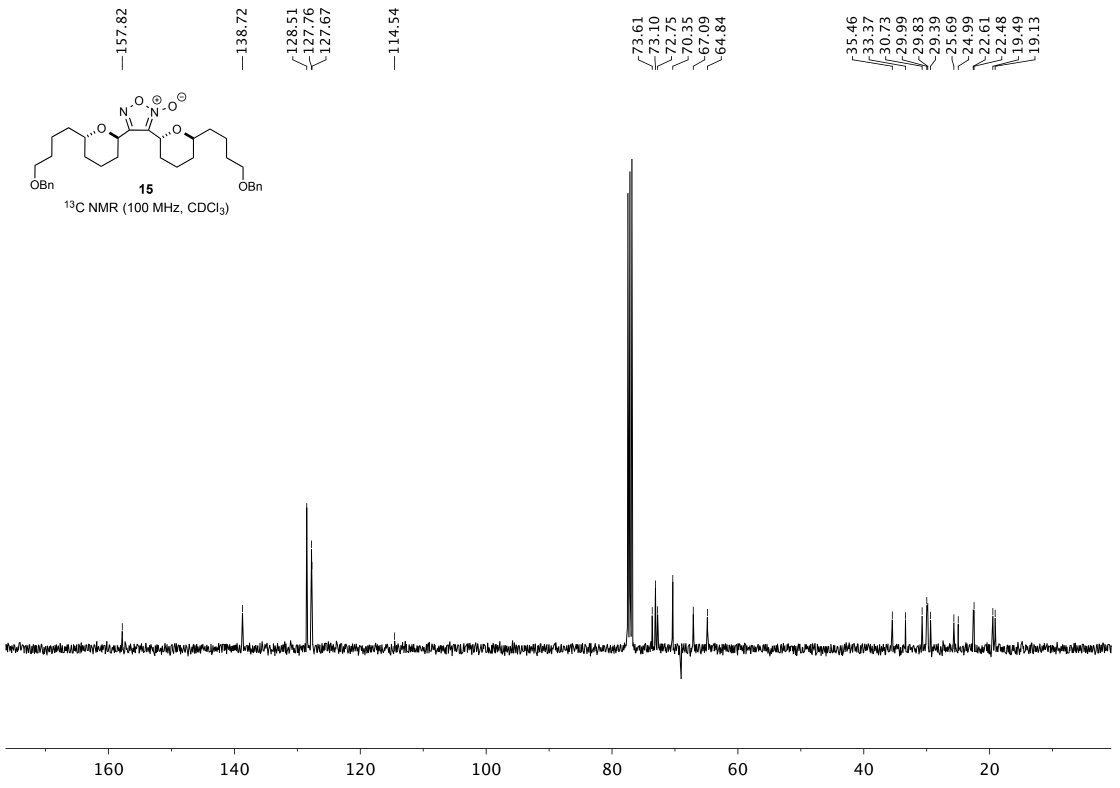
OBn
OBn
15
13C NMR (100 MHz, CDCl3)
157.82 138.72 128.51 127.76 127.67 114.54
73.61 73.10 72.75 70.35 67.09 64.84
35.46 33.37 30.73 29.99 29.83 29.39 25.69 24.99 22.61 22.48 19.49 19.13
160 140 120 100 80 60 40 20

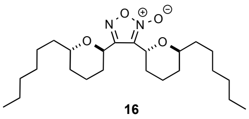

16

^{1}H NMR (400 MHz, $CDCl_3$)

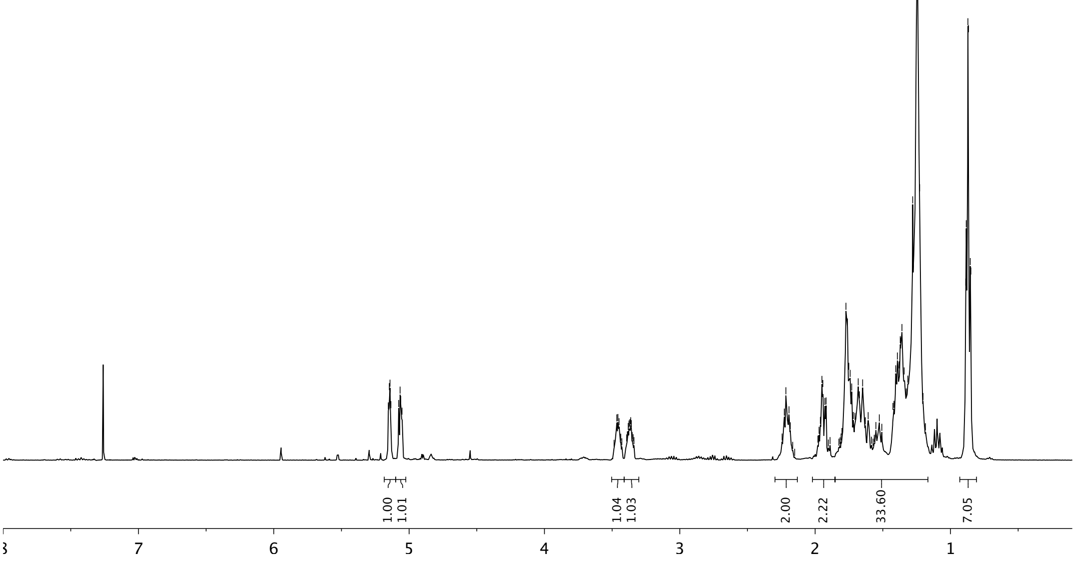

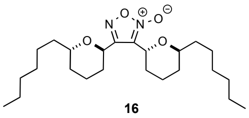

16

^{1}H NMR (400 MHz, $CDCl_3$)

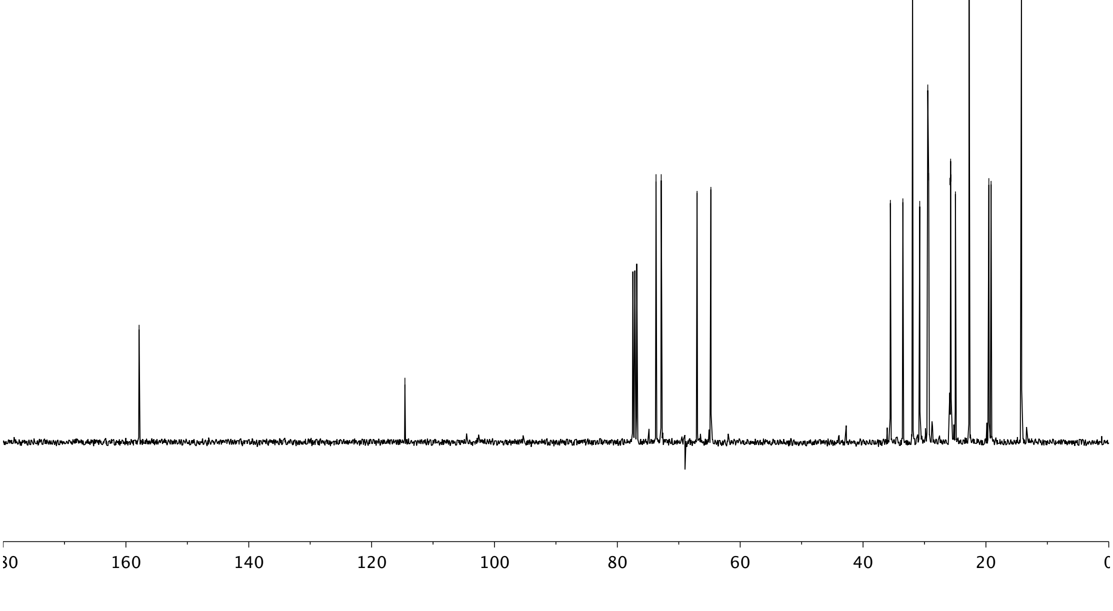

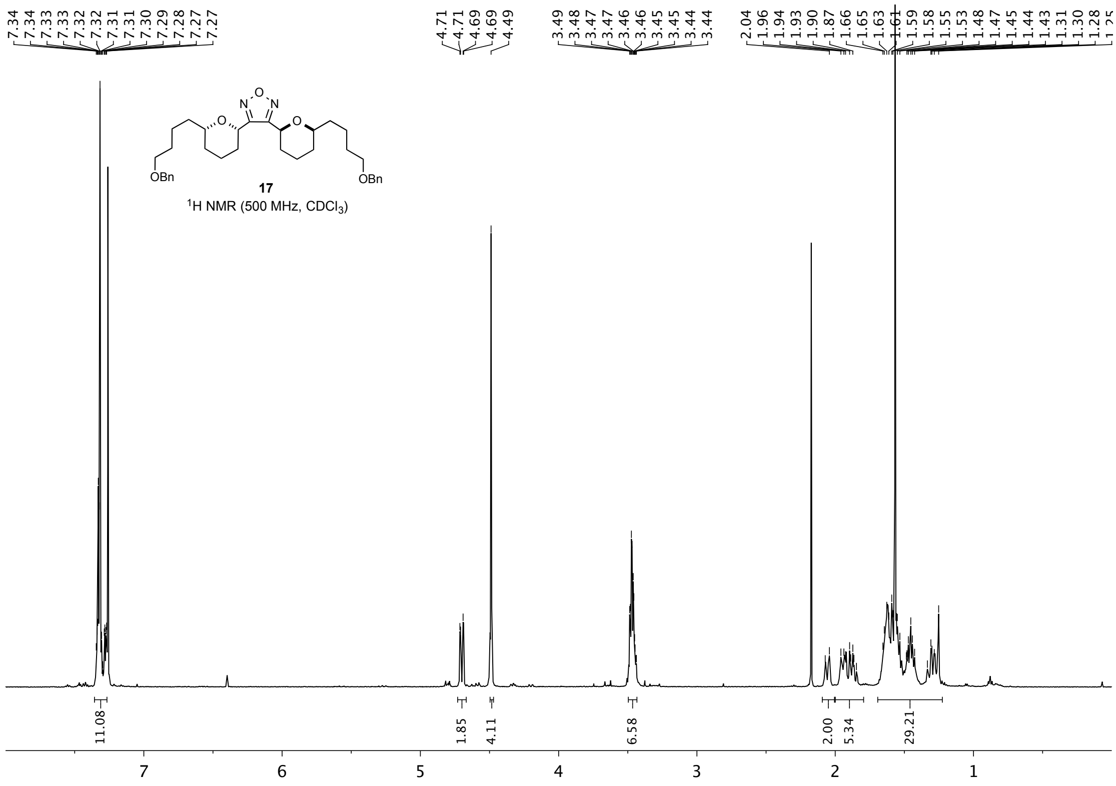
7.34 7.34 7.33 7.33 7.32 7.32 7.31 7.31 7.30 7.29 7.28 7.27 7.27
4.71 4.71 4.69 4.69 4.49
3.49 3.48 3.47 3.47 3.46 3.46 3.45 3.45 3.44 3.44
2.04 1.96 1.94 1.93 1.90 1.87 1.66 1.65 1.63 1.61 1.59 1.58 1.55 1.53 1.48 1.47 1.45 1.44 1.43 1.31 1.30 1.28
OBn
OBn
17
1H NMR (500 MHz, CDCl3)
11.08
1.85
4.11
6.58
2.00
5.34
29.21
7
6
5
4
3
2
1

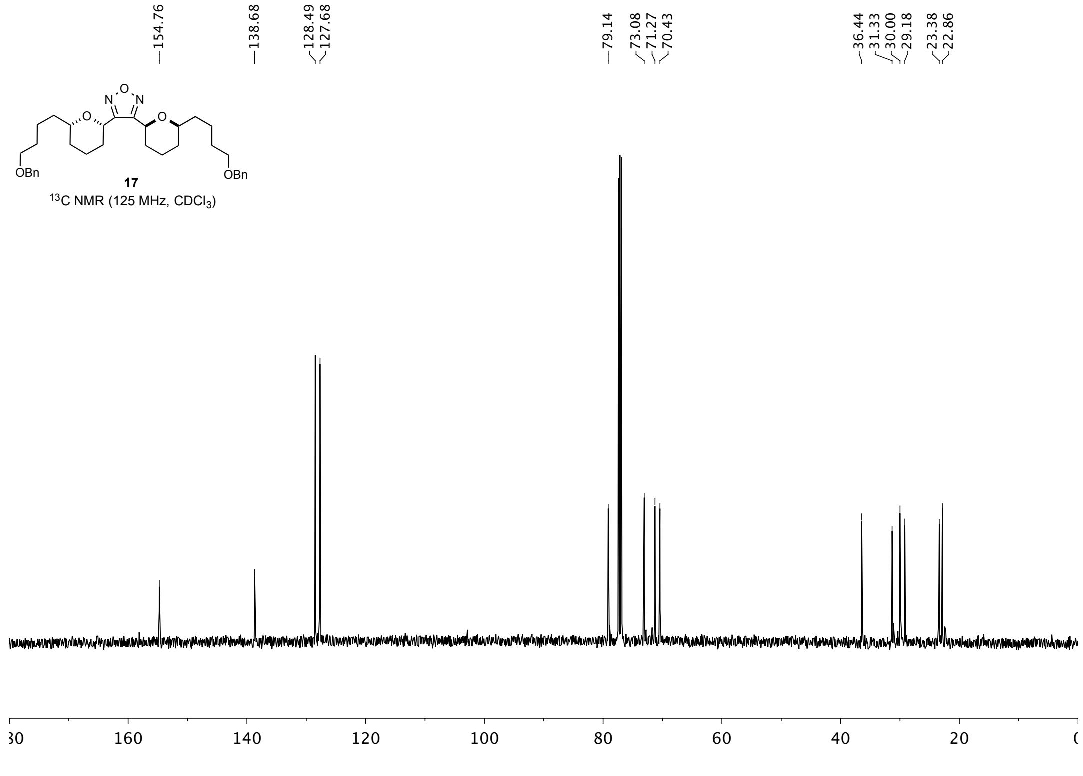
154.76
138.68
128.49
127.68
79.14
73.08
71.27
70.43
36.44
31.33
30.00
29.18
23.38
22.86
OBn
OBn
17
13C NMR (125 MHz, CDCl3)
160
140
120
100
80
60
40
20

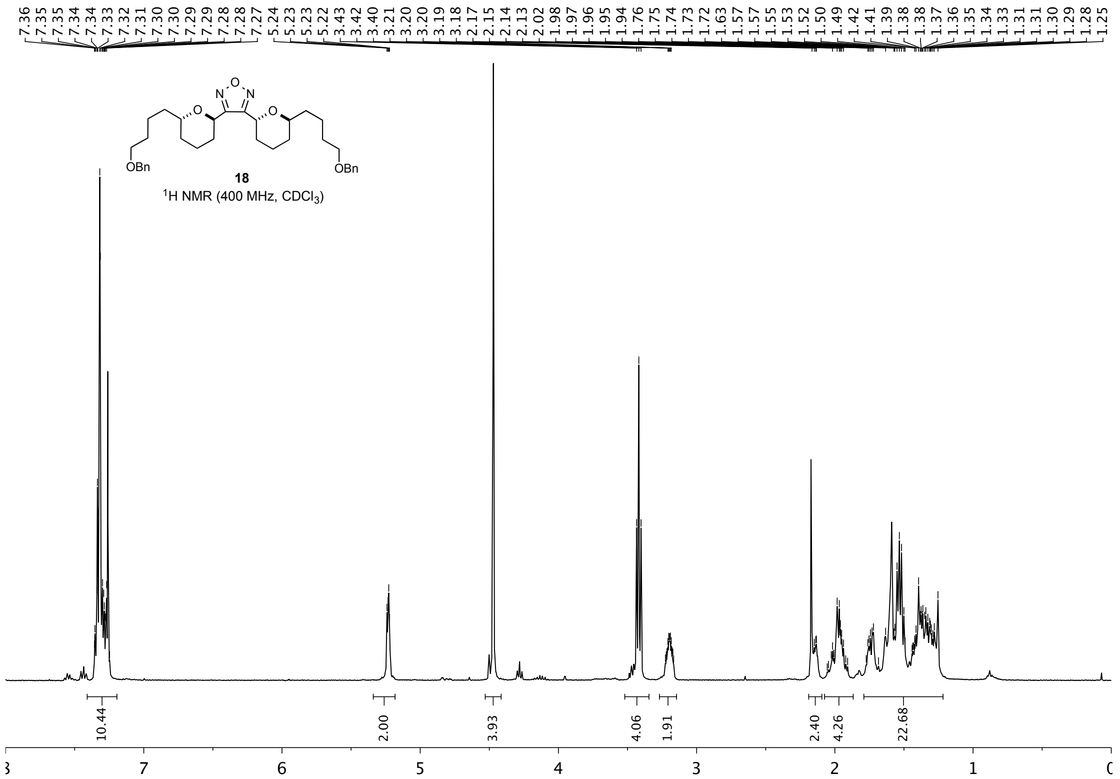
18
^{1}H NMR (400 MHz, CDCl$_3$)

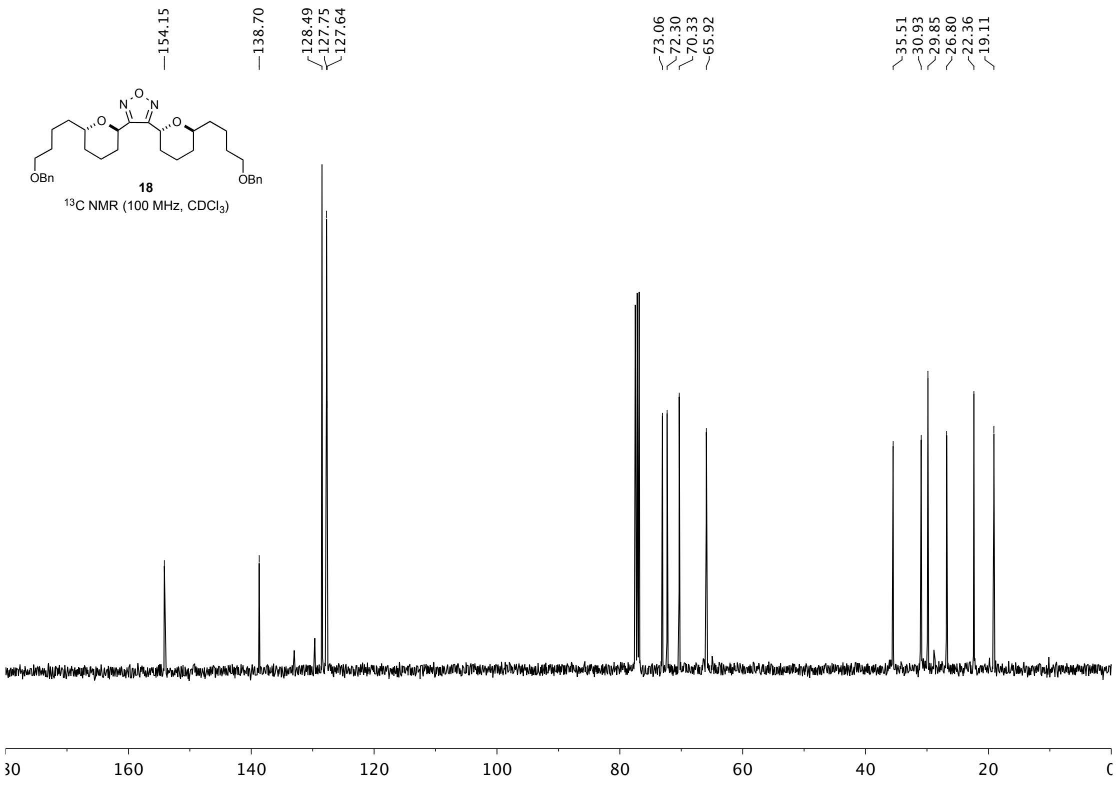
18
^{13}C NMR (100 MHz, CDCl$_3$)

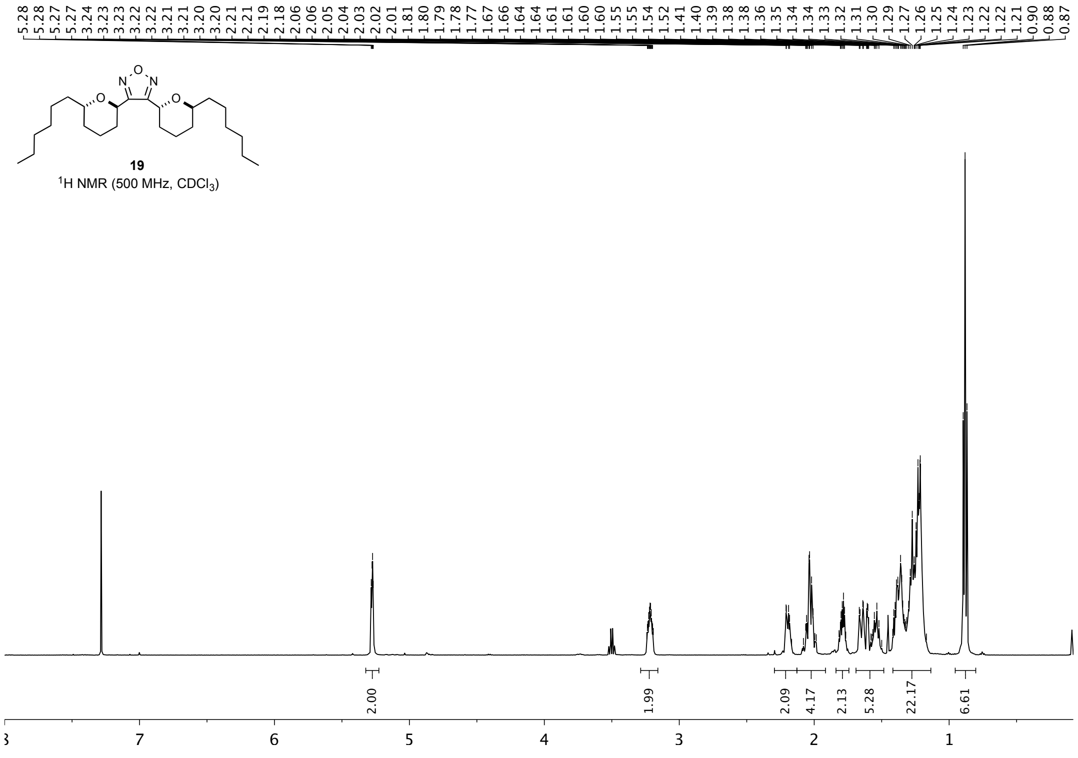

19
^{1}H NMR (500 MHz, CDCl$_3$)

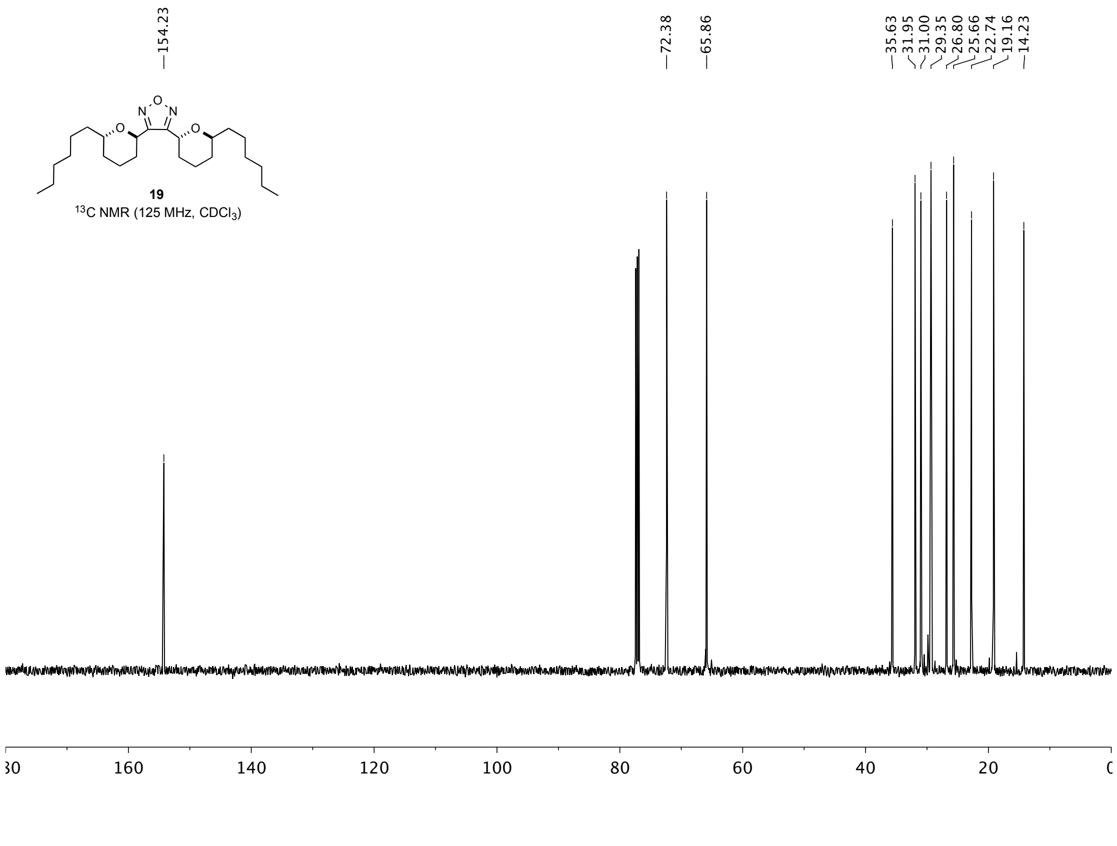

19
^{13}C NMR (125 MHz, CDCl$_3$)
